# Rapid Hypothesis Testing in Candida albicans Clinical Isolates Using a Cloning-Free, Modular, and Recyclable System for CRISPR-Cas9 Mediated Mutant and Revertant Construction

**DOI:** 10.1128/spectrum.02630-21

**Published:** 2022-05-25

**Authors:** Junyan Liu, Amanda K. Vogel, Jian Miao, Jennifer A. Carnahan, David J. Lowes, Jeffrey M. Rybak, Brian M. Peters

**Affiliations:** a Department of Clinical Pharmacy and Translational Science, College of Pharmacy, University of Tennessee Health Science Center, Memphis, Tennessee, USA; b School of Food Science and Engineering, Guangdong Province Key Laboratory for Green Processing of Natural Products and Product Safety, South China University of Technology, Guangzhou, China; c Integrated Program in Biomedical Sciences, College of Graduate Health Sciences, University of Tennessee Health Science Center, Memphis, Tennessee, USA; d Graduate Program in Pharmaceutical Sciences, College of Graduate Health Sciences, University of Tennessee Health Science Center, Memphis, Tennessee, USA; e Pharmacy and Pharmaceutical Sciences Department, St. Jude Children’s Research Hospital, Memphis, Tennessee, USA; f Department of Microbiology, Immunology, and Biochemistry, College of Medicine, University of Tennessee Health Science Center, Memphis, Tennessee, USA; University of Michigan

**Keywords:** CRISPR, Cas9, *Candida*, genetics, molecular mycology, mutant, molecular genetics, mycology

## Abstract

As increasing evidence emerges that interstrain genetic diversity among Candida albicans clinical isolates underpins phenotypic variation compared to the reference isolate SC5314, new genetic tools are required to interrogate gene function across strain backgrounds. Here, the *SAT1*-flipper plasmid was reengineered to contain a C. albicans codon optimized hygromycin B resistance gene (*CaHygB*). Cassettes were PCR-amplified from both *SAT1*-flipper and *CaHygB*-flipper plasmids using primers with homologous sequences flanking target genes of interest to serve as repair templates. Ribonucleoprotein (RNP) complexes containing proprietary CRISPR RNAs (crRNAs), universal transactivating CRISPR RNA (tracrRNA), and Cas9 protein were assembled *in vitro* and transformed, along with both repair templates, by electroporation into C. albicans. Homozygous deletion of the *ADE2* gene results in red-pigmented colonies and this gene was used to validate our approach. Both in SC5314 and a variety of clinical isolates (529L, JS15, SJCA1, TW1), homozygous gene targeting was nearly 100% when plating on media containing nourseothricin and hygromycin B with transformation efficiencies exceeding 10^4^ homozygous deletion mutants per μg of DNA. A gene reversion system was also employed with plasmids pDUP3 and pDIS3 engineered to contain the *ADH1* terminator and an overlap extension PCR-mediated approach combined with CRISPR-Cas9 targeting at the *NEUT5* neutral locus. A variety of single or compound mutants (Δ/Δ*als3*, Δ/Δ*cph1* Δ/Δ*efg1*, Δ/Δ*ece1*) and their revertant strains were constructed and phenotypically validated by a variety of assays, including biofilm formation, hyphal growth, and macrophage IL-1β response. Thus, we have established a cloning-free, modular system for highly efficient homozygous gene deletion and reversion in diverse isolates.

**IMPORTANCE** Recently, phenotypic heterogeneity in Candida albicans isolates has been recognized as an underappreciated factor contributing to gene diversification and broadly impacts strain-to-strain antifungal resistance, fitness, and pathogenicity. We have designed a cloning-free genetic system for rapid gene deletion and reversion in C. albicans clinical isolates that interlaces established recyclable genetic systems with CRISPR-Cas9 technology. The *SAT1*-flipper was reengineered to contain *CaHygB* encoding resistance to hygromycin B. Using a modular PCR-mediated approach coupled with *in vitro* ribonucleoprotein assembly with commercial reagents, both *SAT1*- and *CaHygB*-flipper cassettes were simultaneously integrated at loci with high efficiency (10^4^ transformants per μg DNA) and upward of 99% homozygous gene targeting across a collection of diverse isolates of various anatomical origin. Revertant strains were constructed by overlap extension PCR with CRISPR-Cas9 targeted integration at the *NEUT5* locus. Thus, this facile system will aid in unraveling the genetic factors contributing to the complexity of intraspecies diversity.

## INTRODUCTION

The polymorphic diploid fungus Candida albicans is among the most prevalent human pathogens and responsible for a variety of superficial and invasive mycoses (1). Tractable genetic tools and systems for fungal gene manipulation have played pivotal roles in delineating the molecular mechanisms responsible for drug resistance, metabolic plasticity, physiology, and pathogenic fitness in C. albicans. The earliest approaches relied on chemical or UV-mediated random mutagenesis, which while effective at the time, introduced widespread genomic changes due to associated toxicity (2). The “URA Blaster” technique pioneered improved targeted gene deletion in C. albicans by utilizing a *ura3* auxotroph (e.g., strain CAI4) and cassette containing a copy of the *URA3* gene flanked by direct repeats and regions of target gene homology (3, 4). The cassette could be recycled by plating *URA3* prototrophs on 5-fluoroorotic acid (5-FOA) to spontaneously select for new *ura3* auxotrophs and a subsequent round of transformation required for deletion of the second allele. However, the time required for recycling, associated toxicity of 5-FOA counterselection, and locus-specific effects of *URA3* insertion were problematic.

Creation of strain BWP17 (*ura3*, *his1*, and *arg4* auxotroph derived from reference isolate SC5314) allowed for transformation of gene disruption cassettes containing two of these markers to create homozygous deletion mutants and incorporation of the third containing the remaining marker and wild-type copy of the gene to create an isogenic revertant strain (5). While this process remains highly effective for relatively rapid gene deletion and reversion, it is only applicable to studying gene function in a single strain background. Therefore, approaches utilizing dominant selection markers (e.g., *NAT1*, *SAT1*, *HygB*) to encode resistance to nourseothricin (NAT) or hygromycin B (HygB) have been developed for use in multiple strain backgrounds and clinical isolates (6–8). The “SAT1-flipper” cassette containing a flip recombinase (*FLP*) flanked by short *FLP* recognition target (FRT) sequences has been a particularly powerful tool for recyclable gene deletion in C. albicans (7). Although these techniques and their multiple derivatives have served invaluable roles in better understanding fungal biology in this pathogen, unsatisfactory transformation efficiency and required molecular cloning steps necessitate improved tools.

The advent of clustered regularly interspaced short palindromic repeat (CRISPR-Cas9) related strategies have overcome many of the aforementioned hurdles with respect to genetic manipulation of C. albicans (9). The CRISPR-Cas9 system relies on activity of the bacterial nuclease Cas9 to introduce directed double-strand DNA breaks in the genome of the target organism (10). Inclusion of a repair template containing homologous sequences that flank the cut site can then be incorporated at the targeted locus via homologous recombination. Such repair templates may contain virtually any sequence, including those that encode antimicrobial resistance markers for selection, fluorescent tags, allelic copies from other isolates, or immunodetection tags. The precision of Cas9 cutting is directed by a unique 20 bp protospacer (or guide) sequence that is immediately followed by a protospacer adjacent motif (PAM) site of the typical nucleotide sequence NGG (11). Several highly effective CRISPR-Cas9 systems have been described for use in C. albicans. The first iteration of these systems utilized a C. albicans strain in which the Cas9 coding sequence was introduced into the genome under the constitutive *ENO1* promoter (12). The second transient approach utilized a PCR-mediated strategy to amplify the Cas9 coding and guide sequences that are then cotransformed with the relevant repair template (13). In both cases, promoter and Cas9 coding sequences require optimization for C. albicans in these expression-based systems due to codon usage and expression differences.

Recently, several laboratories have demonstrated that expression-free systems utilizing purified Cas9 and guide RNAs that are assembled *in vitro* to form ribonucleoprotein (RNP) complexes prior to transformation show excellent efficiency for gene deletion in a variety of fungal pathogens, including non-*albicans Candida* species and Aspergillus fumigatus (14, 15). Given the relative straightforward approach, commercial availability, and effectiveness of this system, we leveraged prior gene deletion technologies with the efficiency of CRISPR-Cas9 to develop a new tool for facile gene disruption and reversion in C. albicans. We have created a version of the *SAT1*-flipper that contains a C. albicans optimized hygromycin B resistance (*CaHygB*) cassette (i.e., *CaHygB*-flipper) for use in conjunction with the *SAT1*-flipper to allow for single step, highly efficient homozygous gene targeting using a PCR-mediated approach (6). Transformation with dual-selection markers resulted in a significantly higher percentage of homozygous disruption mutants compared to single markers alone. Cassette excision allowed for marker recycling and generation of compound mutants, including construction of an Δ/Δ*cph1* Δ/Δ*efg1* strain. Additionally, we have created plasmids pDUP3-tADH1 and pDIS3-tADH1 to facilitate construction of revertant strains by introducing alleles at the *NEUT5* neutral locus using a cloning-free, overlap extension PCR strategy. As proof-of-concept using these systems, we successfully deleted and restored the *ADE2*, *ALS3*, and *ECE1* genes in SC5314 or a variety of clinical isolates and validated their phenotypes by assessing pigmentation, biofilm formation, and capacity to elicit IL-1β response in THP-1 macrophages, respectively. Collectively, we anticipate that this modular approach will facilitate rapid construction of mutant and revertant strains to allow for robust hypothesis testing across multiple C. albicans clinical isolates.

## RESULTS

**FIG 1:**
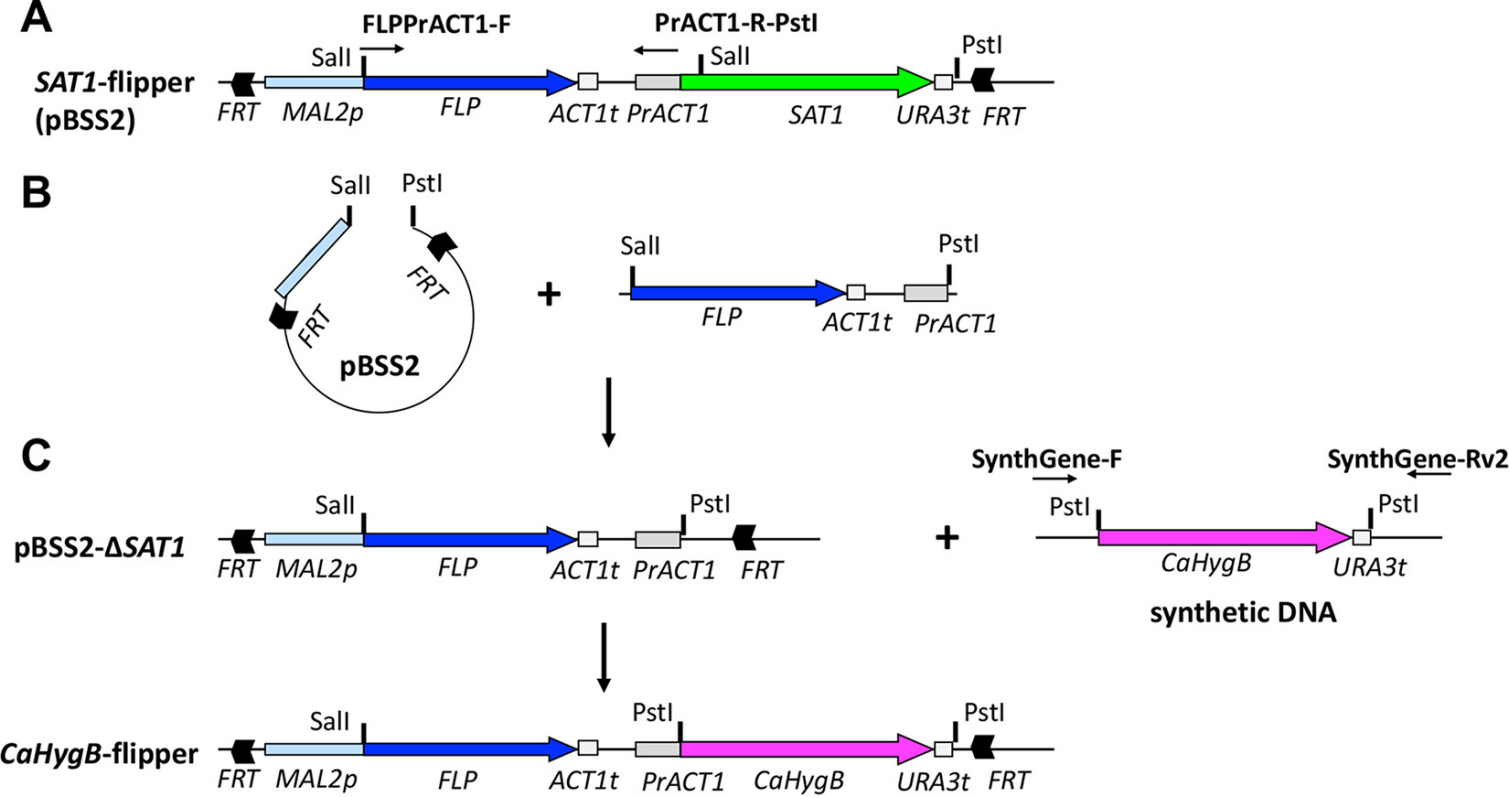
Schematic of *CaHygB*-flipper plasmid construction. (A) A segment containing the flip recombinase (*FLP*), *ACT1* terminator, and *ACT1* promoter of the *SAT1*-flipper plasmid was PCR amplified and (B) ligated into SalI and PstI digested *SAT1*-flipper. This resulted in plasmid pBSS2-Δ*SAT1* which lacked the *SAT1* gene encoding resistance to nourseothricin. (C) A synthetic piece of DNA encoding a C. albicans codon optimized hygromycin B resistance gene (*CaHygB*) and *URA3* terminator was ligated into pBSS2-Δ*SAT1* to create the *CaHygB*-flipper plasmid.

**FIG 2:**
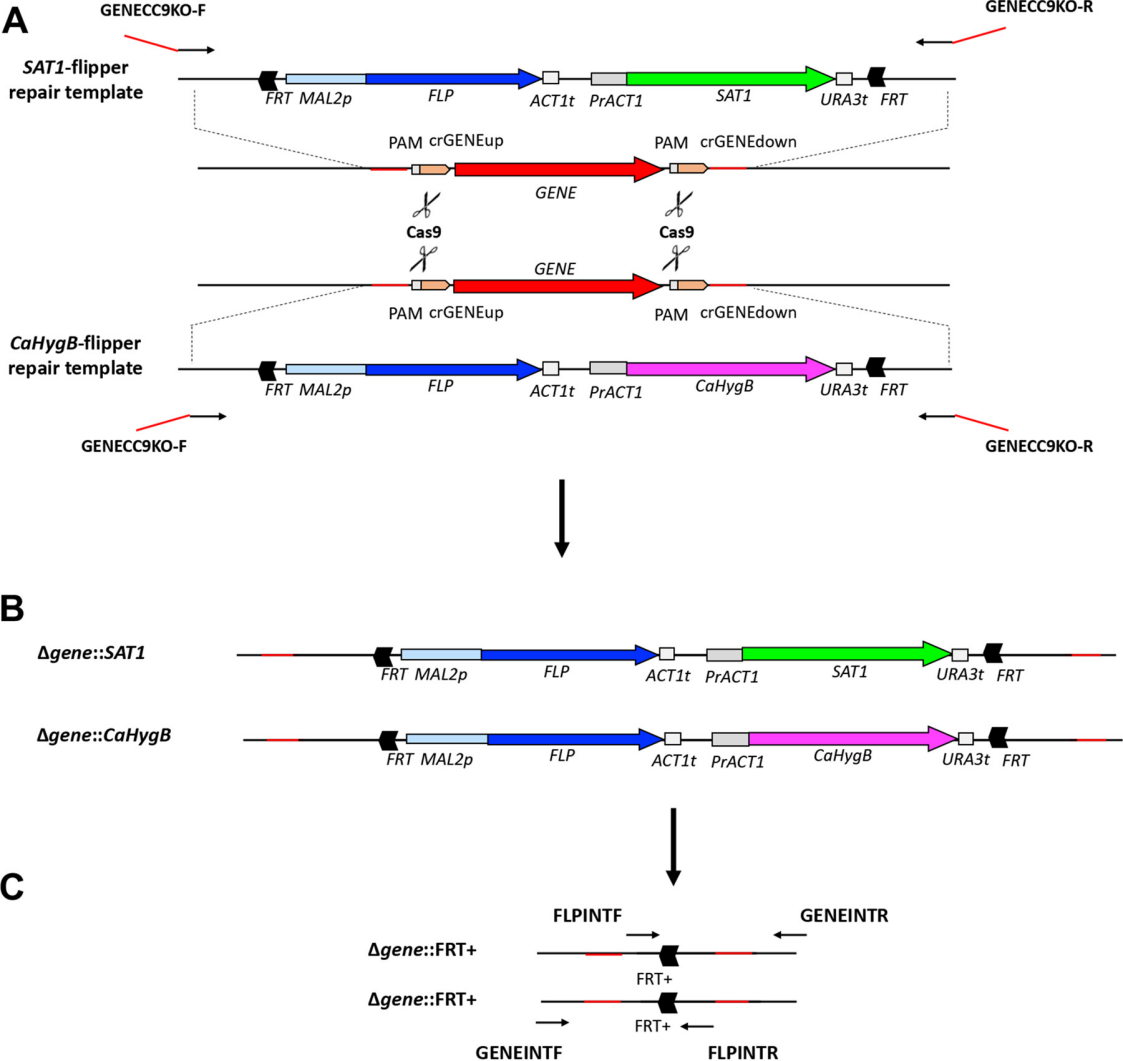
Schematic of CRISPR-Cas9 mediated gene deletion, cassette integration, and excision. (A) GENECC9KO primer pairs are designed to bind to conserved regions on *SAT1*- and *CaHygB*-flipper plasmids (black lines) and also contain gene-specific overhangs (red lines) that are complementary to the target gene of interest and located close to PAM sites in the C. albicans genome that flank the gene targeted for deletion. GENECC9KO primers are used to generate repair templates simultaneously utilized during CRISPR-Cas9-mediated transformation. (B) After transformation and selection on media containing both NAT and HygB, each cassette integrates at each target gene locus to generate homozygous deletion mutants. (C) After growth in maltose medium (YPM), the cassettes are “flipped” out by flip recombinase mediated cleavage at FRT sites. A single FRT site and approximately 200 bp of additional plasmid sequence (termed FRT+) remain at each locus. Primer pairs FLIPINTF and GENEINTR and FLPINTF and FLPINTR are used to detect locus disruption by PCR. Loss of the coding sequence is also confirmed by GENEDET primers.

**FIG 3:**
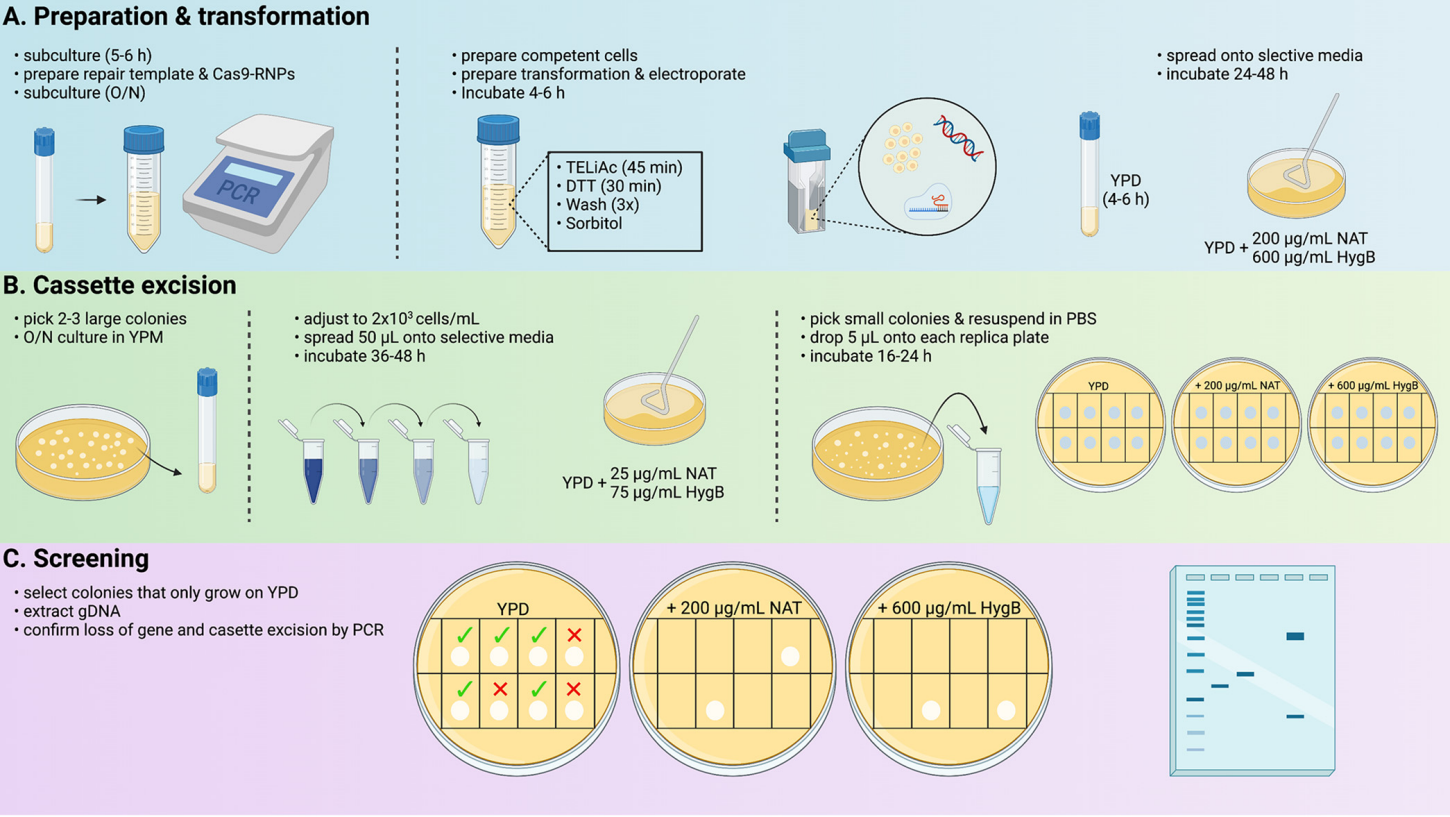
Graphical abstract of CRISPR-Cas9 *in vitro* RNP assembly, gene deletion, and screening protocol. Brief workflow diagram depicting timeline and key steps for high efficiency homozygous mutant and gene revertant construction. Precise details may be consulted in Materials and Methods. Image was created with Biorender.

In order to create a new recyclable dominant selection marker for gene deletion in C. albicans, the *SAT1*-flipper was reengineered to contain a C. albicans codon-optimized hygromycin resistance gene (*CaHygB*) (6, 7). Essentially, this ultimately led to creation of an otherwise isogenic vector termed the *CaHygB*-flipper (Fig. 1). Primers (e.g., GENECC9KO) containing conserved regions with complementarity to both *SAT1*- and *CaHygB*-flippers (Fig. 2A, black arrows), as well as approximately 50 bp overhangs that flank the gene targeted for deletion (Fig. 2A, red lines), were used to amplify regions of these plasmids containing the *MAL2* promoter, *FLP* recombinase, selectable markers, and both FRT sites (Table S1). These PCR products served as repair templates during *in vitro* assembled RNP CRISPR-Cas9 mediated homologous recombination. The general protocol used is summarized in Fig. 3.

**FIG 4:**
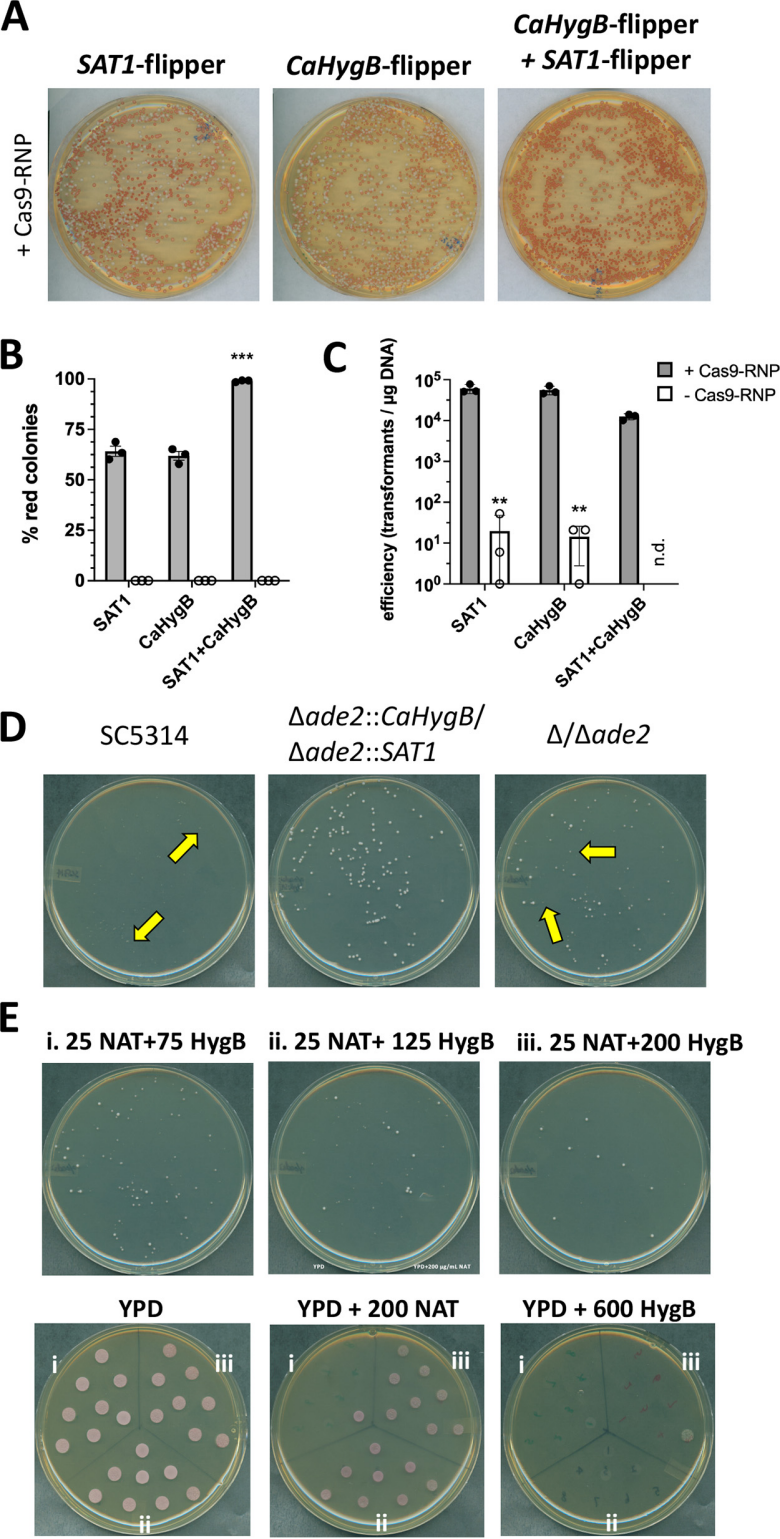
Proof of concept regarding homozygous mutant construction by targeting the *ADE2* locus by *in vitro* assembly of Cas9-RNP complexes. (A) C. albicans strain SC5314 was transformed with or without *in vitro* assembled Cas9-RNPs and *SAT1*, *CaHygB*, or both repair templates targeting the *ADE2* loci and plated onto appropriate selective media. Representative images of each plate were captured by digital scanning. (B) The number of red colonies were counted and divided by total colonies per plate to generate % homozygous targeting values. Data are expressed as the mean ± SD from 3 independent experiments. A one-way ANOVA with Dunnett’s posttest was used for statistical analysis. ***, *P* < 0.001. (C) Transformation efficiency was calculated from 3 independent experiments as described in panel A. A multiple *t* test was used for statistical analysis. **, *P* < 0.01. (D) SC5314, Δ*ade2*::*CaHygB*/Δ*ade2*::*SAT1*, and Δ/Δ*ade2* strains were plated onto YPD medium containing 25 μg/mL NAT and 125 μg/mL HygB and images captured after 1 d of growth by digital scanning. Yellow arrows depict small colonies that struggle to grow under these conditions suggesting susceptibility to both antimicrobials. (E) Strains that presumably underwent cassette excision after growth in YPM were grown on YPD medium containing the indicated concentration of antifungals: (i) 25 μg/mL NAT + 75 μg/mL HygB, (ii) 25 μg/mL NAT + 125 μg/mL HygB, (iii) 25 μg/mL NAT + 200 μg/mL HygB. Individual colonies from each condition above were patched onto YPD containing 200 μg/mL NAT, 600 μg/mL HygB, or no antimicrobial to identify colonies that have underwent excision of both cassettes. Images in all panels are representative of at least 3 independent experiments.

We first aimed to determine whether inclusion of both *SAT1* or *CaHygB* repair templates led to significantly higher percentages of homozygous disruption mutants compared to transformation with a single marker. In order to enumerate this visually, we chose to target *ADE2* for deletion, as loss of both gene copies results in pink to red pigmentation of the colony via build-up of an adenine biosynthetic intermediate, while heterozygous mutants or wild-type colonies remain white (16). Transformation of strain SC5314 with repair templates generated by PCR amplification of *SAT1*-flipper or *CaHygB*-flipper plasmids using primers ADE2CC9KO-F and ADE2CC9KO-R (containing either *SAT1* or *CaHygB* markers alone) and selection on media containing the relevant antimicrobial resulted in approximately 64% and 62% of red pigmented colonies, respectively. Transformation efficiencies were approximately 5 × 10^4^ transformants per μg of DNA (Fig. 4A to C). However, simultaneous transformation with both repair templates and selection on medium containing both antimicrobials resulted in over 99% of red cells, demonstrating that this approach is highly efficient at generating and selecting for homozygous mutants (Fig. 4A-C). Using both markers, homozygous deletion efficiency was calculated to be approximately 1 × 10^4^ transformants per μg of repair template DNA. No Cas9-RNP control transformations resulted in efficiencies that were ≥ 1000-fold lower than when Cas9-RNP was included. Importantly, none of these colonies were homozygous mutants as evidenced by the lack of red pigmentation (Fig. 4A-C).

As the resistance cassettes can be recycled via *FLP*-mediated excision, a confirmed mutant that was genotyped by PCR using SAT1DETF and ADE2INTR and CaHygBDETF and ADE2INTR was grown overnight in yeast peptone maltose (YPM) medium (which drives high level *FLP* expression) alone or similar medium supplemented with NAT and HygB to maintain both markers. The reference isolate SC5314 was similarly grown in medium lacking antimicrobials. The following day, cultures were diluted, plated onto medium containing 25 μg/mL NAT and 125 μg/mL HygB, and colonies observed following incubation for 24 h. As expected, the untransformed reference strain struggled to grow on this medium, forming only small colonies (Fig. 4D). However, the homozygous mutant (Δ*ade2*::*SAT1*/Δ*ade2*::*CaHygB*) that was maintained in NAT and HygB during overnight culture grew almost exclusively as large colonies given that it presumably still contained both resistance markers. Culture in YPM lacking selection resulted in a mixed population of cells of various sizes (some of which were presumably Δ/Δ*ade2*). Images were chosen at an early time point following incubation where pigmentation on YPD medium is not overtly obvious by digital capture (but discernible by eye) as to highlight differences in cell size. Large colonies likely represented those that have not lost a resistance cassette, while very small colonies that resemble growth of SC5314 reasonably indicated loss of both resistance cassettes (Fig. 4D). Small colonies were genotyped by PCR using primer pairs indicated Fig. 2C (e.g., FLPINTF and ADE2INTR and FLPINTR and ADE2INTF), but a surprisingly high number were found to contain an intact resistance cassette and still able to grow on that containing medium, confirming that they had indeed not undergone cassette excision (data not shown).

Therefore, similar experiments were carried out to induce excision of the antimicrobial resistance markers but a range of HygB concentrations (75, 125, 200 μg/mL) were assessed in combination with NAT following growth in YPM. Again, several small colonies were chosen and replica plated onto YPD alone, YPD with 200 μg/mL NAT, or YPD with 600 μg/mL HygB. All strains grew on YPD as expected. However, cells that were originally grown on medium containing 25 μg/mL NAT and 125 μg/mL or 200 μg/mL HygB continued to grow on plates containing high concentration of NAT, indicating that the *SAT1* cassette was maintained (Fig. 4E). Interestingly, these same colonies did not grow on plates containing a high level of HygB. However, cells originally grown on 25 μg/mL NAT and 75 μg/mL HygB largely failed to grow on high concentration selection plates indicating that this concentration was appropriate for identifying cassette excision events. Homozygous mutants that had lost both cassettes were confirmed and genotyped by PCR using a combination of FLPINTF and ADE2INTR, ADE2INTF and FLPINTR, and ADE2DETF and ADE2DETR primers.

**FIG 5:**
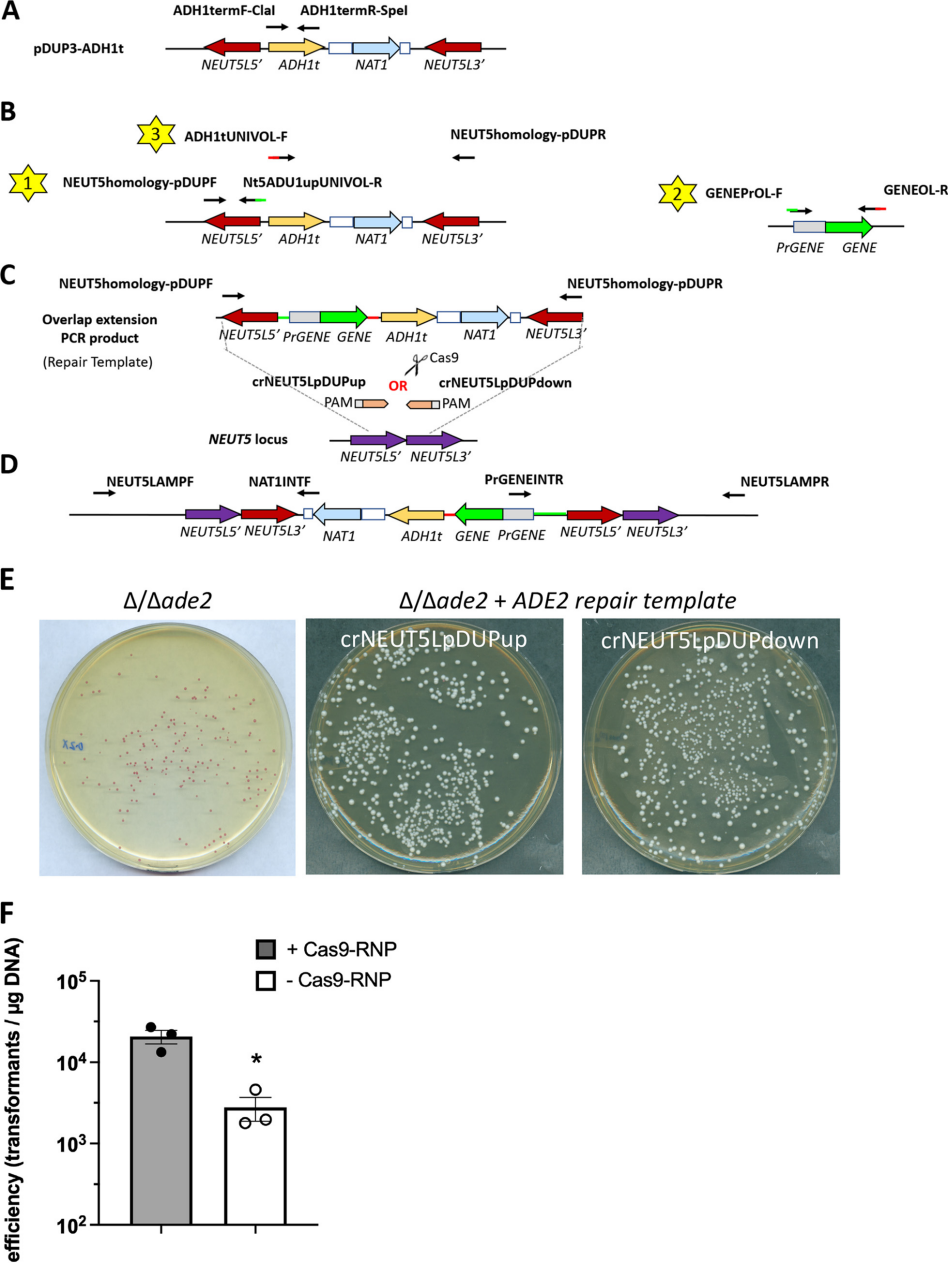
A cloning-free and CRISPR-Cas9-mediated approach to gene reversion at the C. albicans *NEUT5* locus by single-strand crossover. (A) Plasmid pDUP3 was modified to contain the *ADH1* terminator sequence to generate plasmid pDUP3-tADH1. (B) Overlap extension PCR is utilized to fuse 3 PCR fragments generated by independent reactions (yellow stars) using either pDUP3-tADH1 (reactions 1 and 3) or C. albicans genomic DNA (reaction 2) as the templates. Primers contain conserved overlap sequences depicted as red and green lines. (C) The fused cassette is integrated at the *NEUT5* neutral locus by CRISPR-Cas9 transformation in C. albicans using either crNEUT5up or crNEUT5down crRNAs and contains the *NAT1* marker conferring resistance to nourseothricin. (D) The integrated cassette is depicted. Due to position of 5′ and 3′ *NEUT5* homology arms, it integrates in the reverse orientation. Primers NEUT5LAMPF and NAT1INTF and PrGENEINTR and NEUT5LAMPR are used to detect correct integration at the *NEUT5* locus by PCR. (E) The Δ/Δ*ade2* mutant was transformed using the above approach with a wild-type copy of *ADE2*. Digital imaging of YPD plates containing 200 μg/mL NAT depicts reverted white colonies. Images are representative of 3 independent repeats. (F) Transformation efficiency of pDIS3-ADE2 was calculated with (gray box) and without (white box) Cas9-RNP. Data are depicted as the mean ± SD of 3 independent experiments. Statistical significance was assessed using a Student's *t* test. *, *P* < 0.05.

In order to implement a modular and cloning-free system for gene restoration, we next created plasmid pDUP3-tADH1 by inserting the *ADH1* terminator in vector pDUP3 which contains the *NAT1* marker encoding resistance to nourseothricin and integrates by single-strand crossover—a mechanism previously demonstrated to be compatible with CRISPR-Cas9 gene editing (17). The linearized construct inserts into the genome at a site termed the *NEUT5* neutral locus, resulting in duplication of this region (Fig. 5A) (18). Primers were then used to PCR amplify both the 5′ (Fig. 5B, star 1) and 3′ (Fig. 5B, star 3) regions of *NEUT5* that exist in pDUP3. Each of these PCRs generated products with overhangs that are complementary to overhangs found on target gene-specific primers (Fig. 5B, red and green lines with black arrows) used to amplify both the promoter and open reading frame (ORF) of the gene to be restored (Fig. 5B, star 2). After amplification of each of these fragments, an overlap extension PCR was used to stitch together each fragment and the entire fragment then amplified with primers NEUT5homology-pDUPF and NEUT5homology-pDUPR (Fig. 5C). This fragment was then transformed into an Δ/Δ*ade2* strain using our established CRISPR-Cas9 protocol and a single crRNA (crNEUT5LpDUPup or crNEUT5LpDUPdown) to integrate the cassette at the *NEUT5* locus (Fig. 5D) (18). Using both crRNAs at this step disrupts homology regions necessary for recombination at this locus (data not shown). However, use of a single crRNA led to recovery of a large number of previously pink colonies that reverted to white pigmentation when selected on YPD containing 200 μg/mL NAT (Fig. 5E). Integration of the cassette at the *NEUT5* locus was confirmed by PCR using primers NEUT5LAMPF and NAT1INTF and PrADE2INTR and NEUT5LAMPR (Fig. 5D). Presence of the *ADE2* coding sequence was confirmed using primers ADE2DETF and ADE2DETR. Fidelity of the coding sequence was confirmed by amplifying gDNA using primers NEUT5LAMPR and ADH13SEQR and Sanger sequencing this product by using gene specific primers (e.g., ADH13SEQR and ADE2SEQR2). Transformation efficiency for this reaction was approximately 5 × 10^4^ transformants per μg of DNA when Cas9-RNP was included but approximately 8-fold lower in the absence of Cas9-RNP components (Fig. 5F).

A similar approach was undertaken to clone the *ADH1* terminator into vector pDIS3 which contains *NEUT5L* 5′ and *NEUT5L* 3′ homology regions in the same orientation as found in the C. albicans genome (Fig. S1A-E). Thus, this cassette integrates via double-crossover homologous recombination resulting in disruption of *NEUT5L* and forward orientation of the repair template. Integration of the cassette at the *NEUT5* locus was confirmed by PCR using primers NEUT5LAMPF and PrADE2INTR and NAT1INTF and NEUT5LAMPR (Fig. S1D). Presence of the *ADE2* coding sequence was confirmed using primers ADE2DETF and ADE2DETR. Fidelity of the coding sequence was confirmed by amplifying gDNA using primers NEUT5LAMPF and ADH13SEQR and Sanger sequencing using gene specific primers (e.g., ADH13SEQR and ADE2SEQR2). Transformation efficiency was approximately 1 × 10^5^ transformants per μg of DNA and was nearly 1000-fold more efficient when Cas9-RNP was included in the reaction (Fig. S1F).

**FIG 6:**
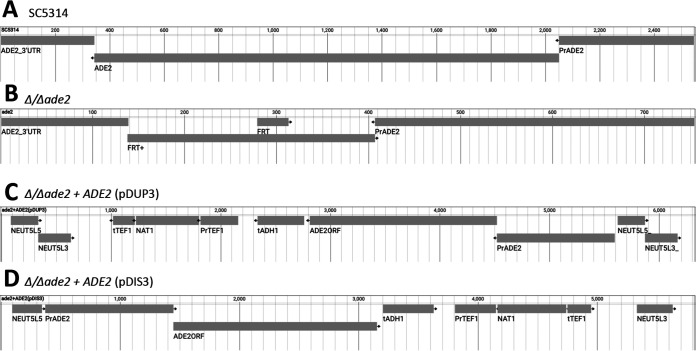
Sequencing reveals on-target deletion of *ADE2* and restoration at the *NEUT5* locus. Genomic DNA from independent transformants of (A) mock-transformed SC5314, (B) CRISPR-Cas9-mediated Δ/Δ*ade2*, or Δ/Δ*ade2*+*ADE2* revertant strains constructed with either (C) pDUP3-tADH1- or (D) pDIS3-tADH1-based repair templates were subjected to PCR using strain-specific primers and amplicons sequenced at Plasmidsaurus. Resulting FASTA files were aligned and visualized using samtools and JBrowse 2 Desktop.

We randomly selected 5 isolates of each of Δ/Δ*ade2*, Δ/Δ*ade2*+*ADE2*, and those that had undergone a mock transformation protocol (electroporation without CRISPR-Cas9 or repair template and plated on nonselective media). Genomic DNA was extracted, targeted PCRs were generated using strain-specific primers, and amplicons sequenced as described. Primer details and analyses are outlined in Materials and Methods. As expected, sequencing reads obtained from SC5314 showed an intact *ADE2* locus. Sequencing of Δ/Δ*ade2* mutants demonstrated that the *ADE2* ORF and partial 5′ and 3′ untranslated regions (UTRs) were replaced by a FRT+ sequence (Fig. 6A and B). Mapping of Δ/Δ*ade2+ADE2* reads confirmed gene restoration at *NEUT5L* in the anticipated manner consistent with pDUP3-tADH1 or pDIS3-tADH1 approaches (Fig. 6C, D). In addition, whole-genome sequencing was performed at a coverage depth ≥ 50× to determine whether our procedure led to any conserved off-target genomic alterations. Further analysis of the Δ/Δ*ade2* strains compared to the mock transformed controls using VCFtools and BLAST did not identify gene variants. Similar comparison of Δ/Δ*ade2* and Δ/Δ*ade2+ADE2* strains demonstrated very few single base changes (total of 8) and none of these were completely conserved across all 5 isolates nor were associated with the *ADE2* locus (Table S2). Therefore, the transformation protocol and CRISPR-Cas9 approach appeared to be highly effective and precise.

**FIG 7:**
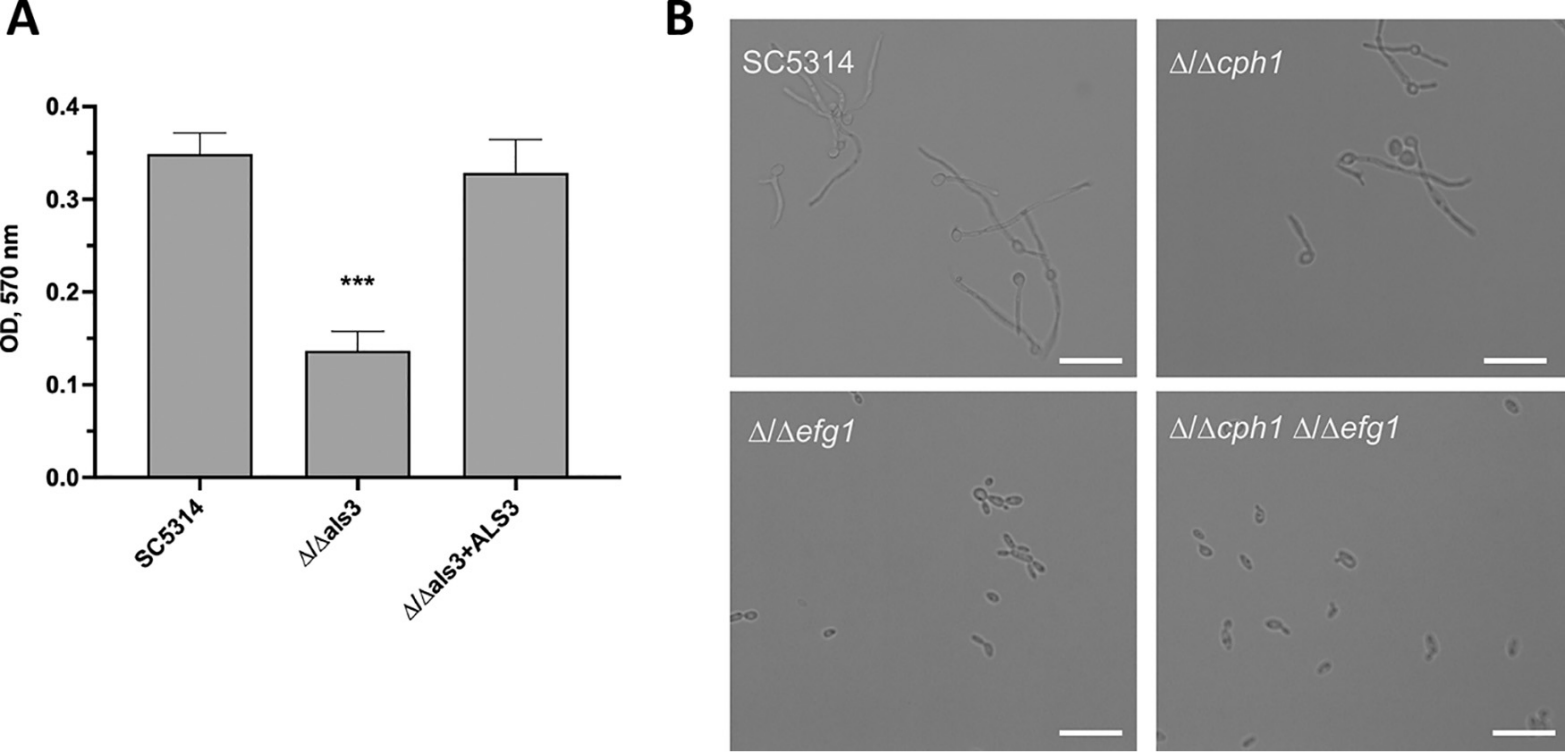
CRISPR-Cas9-mediated homozygous deletion gene targeting and reversion confirms pathogenicity phenotypes and offers the possibility of compound mutant construction. (A) SC5314 and isogenic Δ/Δ*als3* and Δ/Δ*als3*+*ALS3* strains were assessed for their capacity to form biofilm by crystal violet assay and absorbance measured at OD_570_ nm. Data represent the mean + SD. A statistical test was performed using one-way ANOVA and Dunnett’s posttest. ***, *P* < 0.001. (B) Brightfield microscopy mages of SC5314, Δ/Δ*cph1*, Δ/Δ*efg1*, and Δ/Δ*cph1* Δ/Δ*efg1* strains were digitally captured after 4 h of growth in RPMI at 37°C. Images are representative of 3 independent repeats. Scale bar depicts 25 μm.

In order to demonstrate the utility of this system beyond *ADE2* deletion, we deleted several genes in the SC5314 background relevant to C. albicans pathogenicity and hyphal growth using CRISPR-Cas9. Disruption of the hypha-associated adhesin *ALS3* led to significantly reduced biofilm formation on polystyrene as assessed by crystal violet assay, consistent with prior reports (19, 20). Restoration of the *ALS3* coding sequence increased adherence to levels observed with SC5314 (Fig. 7A). In order to demonstrate that the markers are recyclable and the system amenable to multiple gene disruption, we opted to delete the *CPH1* and *EFG1* genes. *EFG1* plays a major role in facilitating the yeast-to-hypha switch and *CPH1* to a lesser role (21, 22). However, deletion of both of these genes severely impacts hypha formation even under conditions that strongly promote filamentation, such as growth in RPMI at 37°C (Fig. 7B). The Δ/Δ*cph1* strain formed filaments similar to SC5314 under this condition, although the Δ/Δ*efg1* strain was unable to do so (Fig. 6B). Subsequent deletion of *EFG1* from the Δ/Δ*cph1* mutant prevented this Δ/Δ*cph1* Δ/Δ*efg1* strain from undergoing the yeast-to-hypha switch and resulted in slightly more ovoid yeast morphology as has been described (21). Thus, recycling of *CaHygB* and *SAT1* markers by cassette excision allows for facile construction of compound deletion mutants.

**FIG 8:**
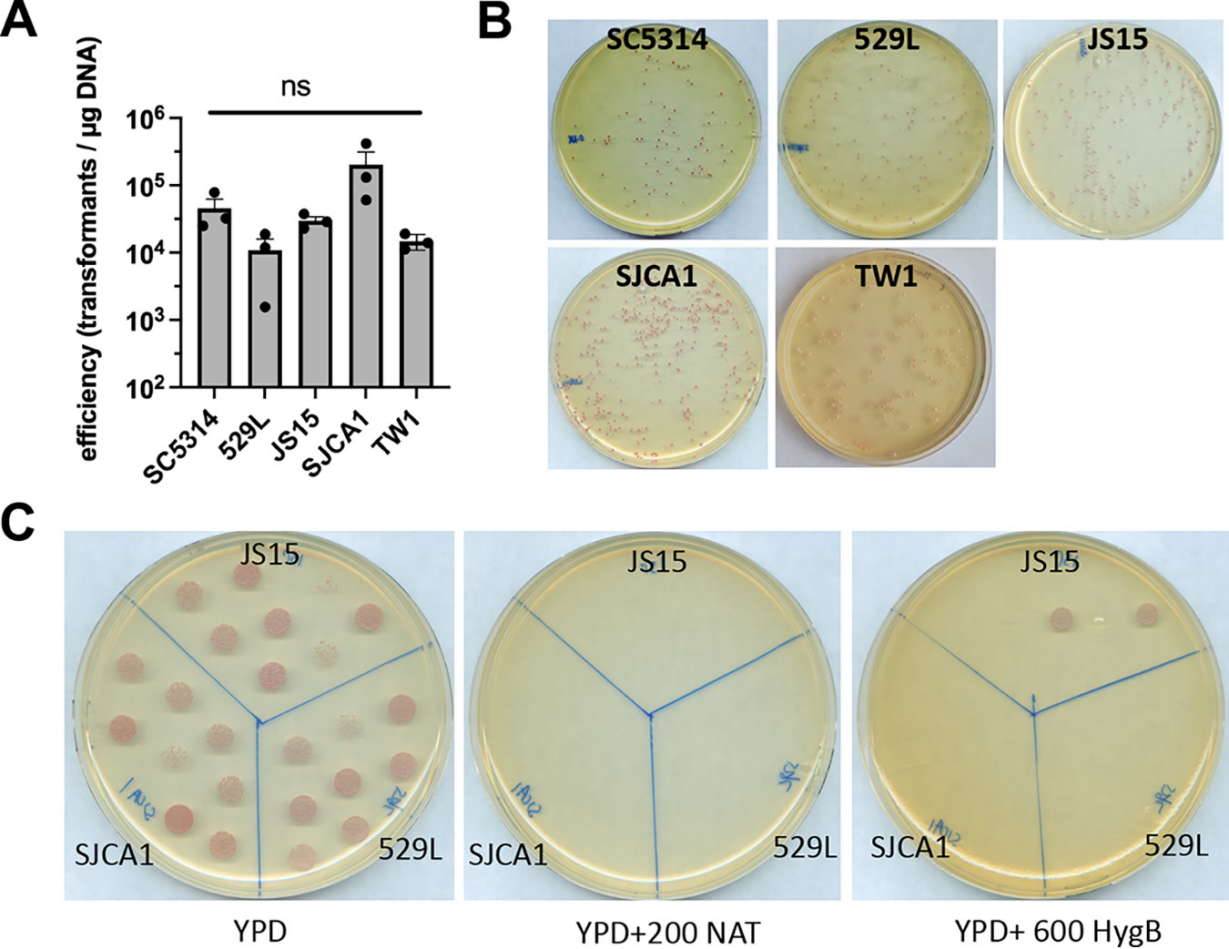
Homozygous gene targeting of the *ADE2* locus using CRISPR-Cas9 is highly efficient across diverse clinical isolates. (A) Transformation efficiency was calculated for strains SC5314 (bloodstream isolate and reference strain), 529L (oral isolate), JS15 (vaginal isolate), SJCA1 (catheter isolate), and TW1 (oral isolate) and plotted as mean ± SD of 3 independent experiments. Data were assessed for statistical significance by one-way ANOVA and Tukey’s posttest. ns, not significant (B) Representative images of YPD plates containing 200 μg/mL NAT and 600 μg/mL HygB were digitally captured. (C) Marker excision for strains JS15, SJCA1, 529L, and TW1 (not shown) were validated by patching resulting colonies after overnight growth in YPM medium and selection onto medium containing 200 μg/mL NAT, 600 μg/mL, or no antimicrobial.

**FIG 9:**
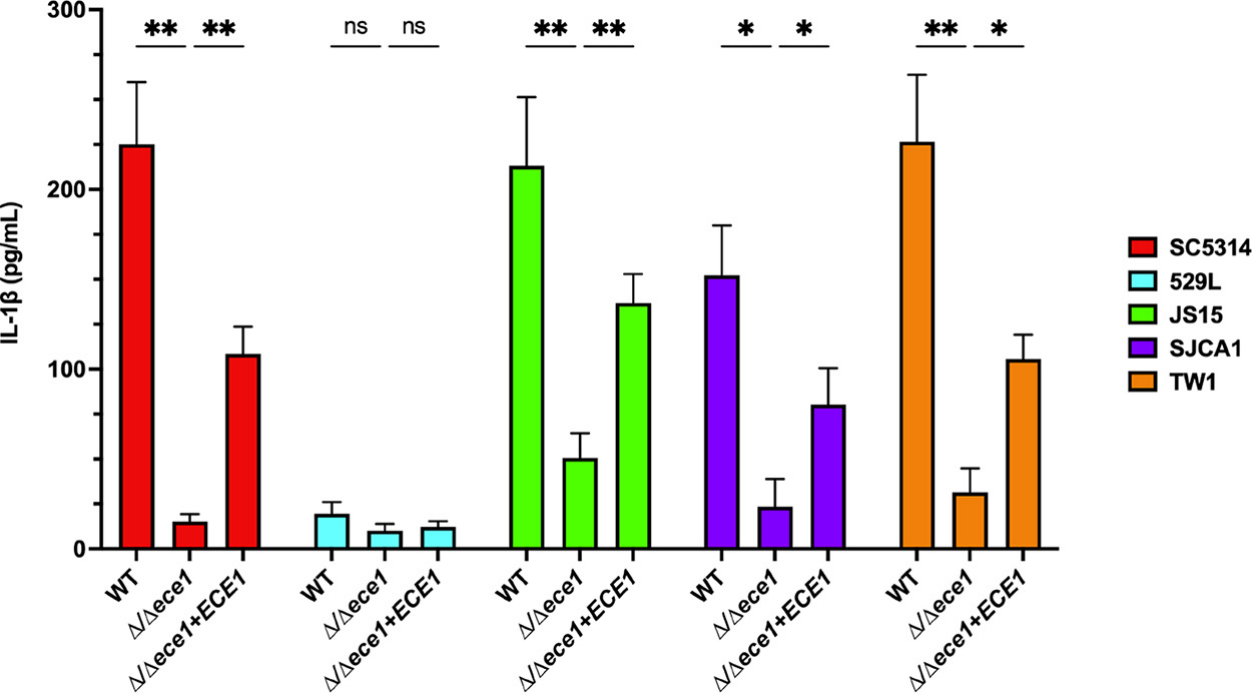
Deletion and restoration of *ECE1* in clinical isolates by CRISPR-Cas9-mediated homozygous gene targeting reveals its conserved crucial role in eliciting IL-1β from THP-1 macrophages. THP-1 cells were challenged with indicated C. albicans wild-type, mutant, and revertant (created with pDUP3-tADH1) strains at an MOI of 5 for 4 h. Culture supernatants were assessed for the inflammatory cytokine IL-1β by ELISA. Data are depicted as the mean + SD from 3 independent experiments. Statistical significance was assessed by multiple one-way ANOVA and Dunnett’s posttests. *, *P* < 0.05; ** *P* < 0.01.

Lastly, we wanted to determine whether this system could be implemented in clinical isolates of C. albicans. Therefore, we utilized isolates of various anatomical origin, including, TW1 (oral), 529L (oral), JS15 (vaginal), and SJCA1 (catheter) and carried out CRISPR-Cas9 mediated deletion of *ADE2* (7, 23, 24). Transformation efficiency was generally similar across isolates (10^4^–10^5^ transformants per μg DNA) and comparable to strain SC5314, although efficiency in strain 529L was slightly lower (Fig. 8A and B). Growth in YPM followed by eventual selective plating on YPD containing 200 μg/mL NAT or 600 μg/mL HygB identified deletion mutants that underwent cassette excision (Fig. 8C). Strains were genotyped by PCR for loss of the resistance cassette and targeted allele for secondary confirmation. In order to validate this approach for assessing pathogenicity across multiple isolates, the *ECE1* gene, which encodes for the peptide toxin candidalysin, was targeted for deletion and restoration (25). Clearly, deletion of *ECE1* in SC5314 and each clinical isolate (except for strain 529L) severely impacted their capacity to elicit IL-1β signaling during challenge of differentiated THP-1 macrophages as previously described (Fig. 9) (26). Restoration of *ECE1* at the *NEUT5* locus with the pDUP3-tADH1-based repair template significantly increased cytokine signaling, but to a lesser extent than the relevant wild-type control (Fig. 9). Restoration of *ECE1* using pDIS3-tADH1 produced a similar phenotype in strain SC5314 (Fig. S2). Strain 529L was previously shown to poorly damage and elicit IL-1β from vaginal epithelium and, so unsurprisingly, deletion or restoration of *ECE1* does not drastically impact macrophage-drive inflammation in this isolate (27, 28). Therefore, this system is highly effective at generating homozygous deletion mutants and revertants across disparate strain backgrounds and its modular design allows for rapid hypothesis testing in clinical isolates.

## DISCUSSION

Recent work by Mitchell and colleagues highlights the extent to which biological information generally regarded as dogmatic for C. albicans has been largely cultivated through extensive analysis of a single reference isolate SC5314 (29). Disruption of key biofilm regulators *EFG1* and *BRG1* severely impacted biofilm formation in both SC5314 and a panel of 4 other diverse clinical isolates, yet disruption of regulators *BCR1* and *UME6* had variable impact depending on strain background. It was ultimately proposed that gene expression “circuits” are diversified based on strain-specific genetic context and that gene function should be validated in multiple strains, along with SC5314, to decipher conserved roles and core regulatory networks. Moreover, traits such as pathogenicity, drug resistance, and metabolic plasticity are well recognized to vary widely among clinical isolates, yet the genetic factors underpinning this diversity is not well-appreciated partially due to lack of robust and facile genetic systems for the manipulation of such strains (23, 28, 30–32).

The approach described herein marries the old with the new, leveraging modern CRISPR-Cas9 technology with established gene disruption techniques to guide construction of deletion mutants across diverse C. albicans isolates originating from a variety of anatomical sites and abiotic surfaces. By utilizing a dual-marker approach to simultaneously target each allele via Cas9 cleavage up- and downstream of the target gene, we were able to achieve > 99% of colonies harboring homozygous deletions with a transformation efficiency of approximately 10^4^ transformants per μg of DNA. While we only quantified these parameters for Δ/Δ*ade2* strains due to clear visual phenotypes, construction of Δ/Δ*als3*, Δ/Δ*efg1*Δ/Δ*cph1*, and Δ/Δ*ece1* mutants were of similarly high efficiency based on genotypic analyses (data not shown). However, efficiency may vary by locus, the impact of gene deletion on basal fitness and growth, or a combination of these factors with genetic background. The ease of use of this system, its modular nature, and the capacity for marker recycling is likely appealing to laboratories with limited exposure to CRISPR-Cas9 technology. As Cas9 and crRNAs are purchased directly from commercial sources, complex design issues and reagent compatibility are addressed by computer-aided ordering systems. Moreover, use of purified Cas9 protein eliminates issues with poor expression from strain- or species-specific promoters or limitations with protein expression due to preferred codon usage. Additionally, the lack of molecular cloning steps and shuttling between bacterial systems, often a bottleneck in strain construction, facilitates rapid engineering of revertant or otherwise “tagged” strains using established selection markers.

There are some disadvantages to our system which may be circumvented by additional vector construction or alternative approaches. Previously described CRISPR-Cas9 systems utilize crRNAs that are synthesized by the target organism and encoded on relatively inexpensive oligonucleotide primers (12, 13). Our system uses universal, but proprietary, crRNAs which are significantly more expensive. However, it is possible that a single crRNA could be utilized to cleave the target gene which would decrease cost per reaction by half. Presumably, this may also decrease deletion efficiency, but we did not explicitly assess this for gene deletion in our studies. A single Cas9-RNP was used for gene restoration at the *NEUT5* locus with efficiencies that paralleled or exceeded those for gene deletion. However, comparatively large homology arms flanking the double-strand break site in these reactions likely promote more efficient repair. Given the single-strand invasion mechanism and *NEUT5* duplication associated with the use of pDUP3-tADH1 repair templates, a relatively high number of transformants were recovered without Cas9. Use of these repair templates is advantageous for achieving multiple integrants, which may be valuable for constructing overexpression or brightly labeled fluorescent strains. However, this potential for copy number variation is likely undesirable otherwise. Use of pDIS3-tADH1 repair templates that integrate via double-crossover homologous recombination and disruption of *NEUT5L*, avoid this complication and high efficiency of transformation was Cas9-dependent. Thus, both approaches have valid use cases and expand versatility of this toolset.

Another potential improvement of this approach would be to develop the capacity to transform strains via established lithium acetate procedures to allow for construction of many strains in parallel and eliminate the need for relatively expensive and time-consuming electroporation procedures (33). Despite our efforts, we were repeatedly unable to achieve high efficiency transformation and selection with CRISPR-Cas9 reagents with lithium acetate protocols extensively used in our laboratory. It is possible that RNPs are unable to penetrate the cell wall without pretreatment (e.g., enzymatic spheroplasting or extended DTT incubation) in the absence of electroporation.

One obvious limitation to revertant construction described here is that the gene of interest is introduced at a neutral locus and not the native one. Should transcriptional accessibility of the native locus differ significantly from *NEUT5* gene expression could be altered. This approach also requires accurate prediction of promoter length to be introduced at the neutral site. While 1 kb of promoter sequence is typically adequate to drive sufficient gene expression in our experience, several genes in C. albicans have lengthy promoter sequences (e.g., *ECE1*) that may extend upward of 10 kb (25, 34). This could explain incomplete complementation of phenotypes observed in our *ECE1* revertant strains. However, gene dosage may be an equally likely explanation. Thus, crRNAs engineered to restore a coding sequence at the native locus may be more desirable in some cases. That said, the crRNAs chosen to direct Cas9 cleavage at *NEUT5* are highly conserved in the bulk of vaginal isolates our laboratory has sequenced (unpublished study) and should work as anticipated across additional isolates. The other more pressing issue with the approach described herein renders the revertant strain permanently resistant to NAT and subsequent gene deletion or protein tagging (e.g., fluorescent reporter) is not readily achievable. It is possible that vectors pDUP3-tADH1 or pDIS3-tADH1 could be reengineered to contain the *CaHygB* cassette for an additional round of transformation and selection on medium containing HygB. Perhaps better would be to engineer pDUP3 to contain contents of the *SAT1*-flipper inclusive of the FRT sites to facilitate integration of the cassette at the *NEUT5* locus yet maintain the capacity to undergo excision.

As the foundation of C. albicans biology is rapidly shifting from an SC5314-centric model to a holistic approach across strains of differing origin, new genetic tools to interrogate gene function across isolates are required. Despite the limitations described above, we have designed and implemented a CRISPR-Cas9 homozygous gene deletion and reversion system that is highly efficient across a diverse set of C. albicans clinical isolates and allows for rapid hypothesis testing to begin unraveling the complexity of intraspecies phenotypic heterogeneity.

## MATERIALS AND METHODS

### Microorganism growth.

C. albicans strains (Table S3) were maintained as glycerol stocks stored at –80°C. A small amount of stock was streaked onto yeast-peptone dextrose (YPD) agar and incubated at 30°C for 24–48 h to obtain isolated colonies. A single colony was transferred to liquid YPD medium and incubated at 30°C with shaking at 200 rpm overnight unless noted otherwise.

### Construction of the *CaHygB*-flipper plasmid.

BglI digested, phosphatase-treated, and heat-inactivated pBSS2 plasmid containing the *SAT1*-flipper disruption cassette from pSF2 was used as the template for a PCR with primers FLPPrACT1-F and PrACT1-R-PstI (Fig. 1A) (7, 35). This reaction generated a fragment containing the flip recombinase coding sequence (*FLP*), *ACT1* terminator (*ACT1*t), and *ACT1* promoter (Pr*ACT1*) which was then purified over a column (ThermoScientific) and digested with SalI and PstI. The *FLP*-*ACT1*-Pr*ACT1* fragment was then ligated into SalI and PstI digested pBSS2 to generate plasmid pBSS2-Δ*SAT1* which was subsequently transformed into Escherichia coli DH5α (Fig. 1B). Accuracy of the *FLP* coding sequence was verified by Sanger sequencing using primers CaPrMAL2-SEQF and FLP-SEQF. A synthetic DNA gBlock containing a C. albicans codon optimized hygromycin resistance (*CaHygB*) gene from E. coli and C. albicans *URA3* terminator (URA3t) sequence flanked by PstI restriction sites and amplification tags was designed (Integrated DNA Technologies [IDT]) (6). The gBlock was PCR amplified using primers SynthGene-F and SynthGene-Rv2, reaction product column purified, digested with PstI, and ligated into PstI-digested pBSS2-Δ*SAT1* to yield plasmid *CaHygB*-flipper, which was subsequently transformed into DH5α (Fig. 1C). Correct directionality of the inset was verified by PCR amplification with primers CaPrACT1-DETF and CaHygB-DETR. Fidelity of the *CaHygB* coding sequence was confirmed by Sanger sequencing (Genewiz) using primers UNIVCRISPR-R and CaHygB-SEQR. The resulting *CaHygB*-flipper is essentially an exact mimic of pBSS2 with the *SAT1* coding sequence replaced by the *CaHygB* gene (Fig. 1A and C). Plasmids pBSS2 and CaHygB-flipper were verified by sequencing (plasmidsaurus.com).

### Selection of short CRISPR RNA (crRNA) sequences.

Protospacer sequences of 20 nucleotides (nt) occurring next to CGG, AGG, or TGG protospacer adjacent motif (PAM) sites were identified in 5′ (crGENEup) and 3′ (crGENEdown) regions either flanking or at distal ends of the target gene coding sequences. Sequences with > 15 nt similarity at off-target sites were excluded. The Eukaryotic Pathogen CRISPR guide RNA/DNA Design Tool (http://grna.ctegd.uga.edu) was used to aid and confirm guide selection using Streptococcus pyogenes Cas9 (SpCas9) and the C. albicans SC5314 genome sequence as search parameters (36). Selected guide sequences were also searched (http://blast.ncbi.nlm.nih.gov) against the C. albicans SC5314 genome to confirm lack of potential off-target integration. Guide RNA sequences were ordered from IDT by supplying the protospacer sequence in the 5′ to 3′ direction excluding the adjacent PAM site. The full crRNA was generated by IDT which contains additional nt sequences that are complementary to a proprietary universal transactivating CRISPR RNA (tracrRNA) sequence used in all CRISPR-mediated transformations.

### Generation of gene disruption repair templates.

Primers were designed that contained sequences complementary to both the *SAT1*-flipper and *CaHygB*-flipper plasmids just outside both minimal flip recombinase inverted repeat sites (FRT). These sequences remained constant for generation of all disruption cassettes (Fig. 2A, black arrows). These primers also contained 40–50 bp homology arms complementary to sequences flanking the targeted deletion (Fig. 2A, red bars) and are within approximately ≤ 30 bp outside the relevant crRNA binding site. Primers were named GENECC9KO-F (Gene CRISPR-Cas9 Knockout) and GENECC9KO-R to indicate orientation. Phusion high fidelity polymerase (ThermoFisher) and relevant GENECC9KO primer pairs were used to PCR amplify BglI-digested and phosphatase-treated *SAT1*-flipper and *CaHygB*-flipper plasmids to generate products of 4.4 kb and 4.2 kb, respectively, according to manufacturer’s directions. Reactions contained 1× HiFi Buffer, 1 μL of 1:100 diluted template DNA, 2.5 μL of each primer, 0.3 mM dNTPs, 2 mM MgSO4, and 0.5 μL of Phusion polymerase in a 50 μL reaction volume. Reaction times consisted of a single extended denaturation step at 98°C for 30 s, followed by 30 cycles of denaturation at 98°C for 10 s, annealing at 63.7°C for 20 s (according to ThermoFisher Calculator), and extension at 72°C for 2.5 min. A final extension for 5 min at 72°C was also performed. Products were cleaned by column purification prior to transformation and quantitated by A260/A280 readings via NanoDrop.

### *In vitro* assembly of Cas9-ribonuceloprotein (RNP) complexes.

Both crRNAs and tracrRNA were resuspended in supplied nuclease-free Duplex Buffer (IDT) to 100 μM prior to use and stored at –20°C. Duplexes for each crRNA were prepared by combining 1 μL of tracrRNA, 1 μL of a single crRNA, and 23 μL of nuclease-free water. Reaction mixtures were incubated at 95°C in a thermocycler for 5 min, chilled on ice for 10 min, and then kept at room temperature. RNP complexes were created by combining 3.6 μL of each crRNA-tracrRNA duplex with 2 μL of Alt-R SpCas9 nuclease V3 (IDT) previously diluted 1:10 in nuclease-free water (2 μg Cas9 total per reaction) and incubated at room temperature for at least 5 min.

### CRISPR-Cas9 mediated transformation.

Our approach was adapted from Grahl, et al. with some modifications (15). A single colony of each C. albicans background strain was inoculated in 1 mL of YPD broth and shaken overnight at 200 rpm at 30°C. The next day, cells were gently vortexed to create a homogeneous suspension and 5 μL of cells transferred to 2 mL YPD and cultured as above for approximately 6 h. A 50 μL aliquot was transferred into 25 mL of YPD in a 50 mL conical tube and shaken overnight at 200 rpm at 30°C. The following day, cells were centrifuged at 4000 rpm for 5 min and the pellet gently resuspended in 10 mL transformation buffer (1× Tris-EDTA [Fisher, Cat number BP2475500, 0.1 M lithium acetate {LiAc}], pH 7.5). Cells were returned to the 30°C incubator with shaking for approximately 45 min. Immediately following, 250 μL of 1 M dithiothreitol (25 mM final) was added and cells returned to the 30°C shaking incubator for exactly 30 min. Cells were diluted with 40 mL of ice-cold distilled water and centrifuged at 4000 rpm for 5 min. The supernatant was discarded, and the cells were resuspended in 25 mL of ice cold distilled water, followed by centrifugation. Cells were then resuspended in 5 mL of ice-cold 1 M sorbitol and centrifuged for 5 min as above. The supernatant was discarded by decanting, the pellet resuspended in the residual liquid, and stored on ice. Aliquots of 40 μL were transferred to chilled 0.2 cm gap electroporation cuvettes. Transformation reactions also received 9.2 μL of RNP complex and approximately 1 μg of PCR-generated repair template. No-DNA control transformation reactions received an equivalent volume of nuclease-free water. Cells were pulsed a single time at 1.8 kV using a Gene Pulser (Bio-Rad). Cells were immediately diluted in 1 mL of ice-cold 1 M sorbitol, transferred to an Eppendorf tube, and centrifuged at 4000 rpm for 1 min. Supernatants were removed by pipetting, 1 mL of YPD used to gently resuspended the cells by gentle stirring with minimal pipetting, and transferred to snap-cap tubes. Tubes were incubated with shaking for 4–6 h at 30°C. Cultures were centrifuged at 4000 rpm for 1 min, supernatant removed by decanting, and pellet resuspended in 1× TE buffer. Several semilog dilutions were prepared in TE buffer prior to spread-plating 100 μL of each suspension on YPD plates containing a range of nourseothricin (75 – 200 μg/mL, GoldBio) and hygromycin B (400–700 μg/mL, GoldBio). Plates were incubated at 30°C for 24–48 h to observe colony growth. Cassette integration at the locus of interest is depicted in Fig. 2B.

### *SAT1*- and *CaHygB*-flipper cassette excision and confirmation.

Colonies growing on YPD plates containing optimized concentrations of nourseothricin (200 μg/mL) and hygromycin B (600 μg/mL) indicated successful integration of both cassettes. Selected colonies were cultured overnight in yeast-peptone (YP) medium containing 2% maltose (YPM) to induce cassette excision as the flip recombinase is drive by the maltose-responsive *MAL2* promoter. The following day, cells were washed twice by centrifugation in sterile PBS, counted by hemocytometer, and diluted to approximately 2 × 10^3^ cells/mL. Aliquots (50 μL) were spread onto YPD agar plates containing 25 μg/mL nourseothricin and a range of hygromycin concentrations (75–200 μg/mL) and incubated for 24 h at 30°C. Comparatively small colonies were chosen as they purportedly indicated loss of the *SAT1* and Ca*HygB* resistance cassettes, resuspended in 15 μL of sterile PBS, and 3 μL drops placed onto YPD alone, YPD + 200 μg/mL nourseothricin, and YPD + 600 μg/mL hygromycin to confirm susceptibility after incubation at 30°C for 24 h. A 3 μL aliquot from the previously resuspended colony was used to start an overnight culture in YPD medium and gDNA extracted as described previously. Correct genomic integration and excision of the resistance cassettes were confirmed by PCR amplification of extracted gDNA using primers FLPINTF and GENEINTR and FLPINTR and GENEINTF (37). FLPINT primers bind in the “scar” (termed FRT+) left behind after cassette excision and GENEINT primers bind in genomic regions flanking the integration site (Fig. 2C). Loss of the gene coding sequence was confirmed by a second PCR using primers GENEDETF and GENEDETR which bind within the targeted deletion. Genomic DNA isolated from the original untransformed strain was used as a positive control. Thus, lack of a band using GENEDET primers and presence of the appropriately sized band with FLPINT and GENEINT primers confirmed the desired genotype.

### Construction of plasmids pDUP3-tADH1 and pDIS3tADH1.

NheI-linearized and phosphatase-treated vector pKE4 was used as the template in a PCR with primers ADH1termF-ClaI and ADH1termR-SpeI to amplify the *ADH1* terminator (*ADH1*t) (Fig. 5A and Fig. S1A) (38). Vectors pDUP3 (integrates at the C. albicans neutral locus [*NEUT5L*] by single-strand crossover resulting in *NEUT5* duplication) and pDIS3 (integrates at *NEUT5* by homologous recombination resulting in *NEUT5* disruption) were digested with ClaI and SpeI and the similarly digested *ADH1*t fragment were ligated together and transformed into DH5α (18). The resulting plasmids were named pDUP3-tADH1 and pDIS3-tADH1, respectively. Plasmids were verified by sequencing (plasmidsaurus.com).

### Cloning-free construction of isogenic revertant strains at the *NEUT5* locus.

Revertant construction was achieved by overlap extension PCR from three initial PCRs that ultimately served as the repair template during CRISPR-Cas9 mediated transformation (39). All reactions used SuperFi II high fidelity polymerase (ThermoFisher) designed to use an invariant annealing temperature of 60°C. The first and third PCRs remained constant for each revertant and used PmlI-linearized and phosphatase-treated pDUP3-tADH1 or pDIS3-tADH1 as the template. The first reaction used primers NEUT5homology-pDUPF or NEUT5homology-pDISF and N5ADHup-UNIVOLR to amplify the NEUT5L5’ region. The third reaction used primers ADH1tUNIVOL-F and NEUT5homology-pDUPR or NEUT5homology-pDISR to amplify the NEUT5L3’ region. Primers N5AHDup-UNIVOLR and ADH1UNIVOL-F contain unique homology arms at the 3′ (Fig. 4B and Fig. S1B, green and black arrow) and 5′ ends (Fig. 4B and Fig. S1B, red and black arrows), respectively, that are complementary to homology arms used in the second PCR. The second PCR used gene-specific primers PrGENEOL-F and GENEOL-R to amplify C. albicans genomic DNA (complementary homology arms noted with red and green arrows, Fig. 4B and Fig. S1B). All PCRs were confirmed for appropriately sized products by agarose gel electrophoresis, cleaned by column purification, and quantitated by NanoDrop A260/280 values. An overlap extension PCR containing 10 μL SuperFi II mastermix and equimolar amounts of each PCR (100 ng of the longest fragment) was incubated for 15 cycles with the following conditions: 10s at 98°C, 10s at 60°C, and 3 min at 72°C. Importantly, no primers were added at this step. A subsequent PCR was ran using 25 μL SuperFi II master mix, 5 μL of overlap extension product, 2.5 μL each of NEUT5homology-pDUPF and NEUT5homology-pDUPR or NEUT5homology-pDISF and NEUT5homology-pDISR primers, and water for 30 cycles using similar conditions as above; the fused product is as depicted in Fig. 4C and Fig. S1C. The resulting fragment was confirmed by gel electrophoresis, cleaned by column purification, and quantitated by NanoDrop. The same protocol for CRISPR-Cas9 mediated transformation was followed as described previously, except crRNAs (pDUP3-based: crNEUT5pDUPup or crNEUT5pDUPdown; pDIS3-based: crNEUT5pDISup or crNEUT5pDISdown) were utilized that direct the overlap extension repair template for integration at the *NEUT5* locus (18). A single-strand invasion mechanism drives integration of pDUP3-based repair templates, resulting in duplication of the NEUT5 locus and reverse orientation of the cassette (Fig. 4D). Integration of pDIS3-based repair templates occurs via homologous recombination, disrupting the *NEUT5* locus and integration in the forward orientation (Fig. S1D). Transformants were plated onto YPD containing 200 μg/mL nourseothricin, grown overnight in YPD, and gDNA extracted. PCR amplification of gDNA using primers NAT1INTF and NEUT5LAMPF and PrGENEINTR and NEUT5LAMPR were used to confirm pDUP3-based cassette integration. Primers NEUT5LAMPF and PrGENEINTR and NAT1INTF and NEUT5LAMPR were used to confirm integration of pDIS3-based repair templates. A subsequent PCR using GENEDETF and GENEDETR primers was used to confirm gene restoration; gDNA from the original strain served as the positive control and that from the isogenic deletion mutant served as the negative control. Accuracy of each gene-specific coding sequence was confirmed by amplification of gDNA using high fidelity polymerase and NAT1DETR and NEUT5LAMPR or NEUT5LAMPF primers and confirmed by Sanger Sequencing with additional GENEDET, GENESEQ, and/or ADH13SEQR primers.

### Imaging of agar plates.

A digital camera or scanner was used to capture images of agar plates either immediately following incubation at 30°C or following a resting period of 2–3 d at 4°C to allow for robust red pigmentation.

### Calculation of homozygous deletion and transformation efficiency.

In order to determine recovery of cells harboring homozygous gene deletions, transformation with the *ADE2* disruption cassettes containing the *SAT1* marker alone, *CaHygB* marker alone, or both was performed with or without Cas9-RNP, diluted, and plated on selective medium containing, 200 μg/mL nourseothricin, 600 μg/mL hygromycin B, or both antimicrobials, respectively. The number of pink and white colonies were expressed as the total percentage of colonies recovered (approximately 5–500 colonies per plate depending on dilution and reaction). Following the transformation procedure for *ADE2* disruption using dual repair templates, reaction mixtures were diluted in a semilog fashion prior to plating on YPD + 200 μg/mL nourseothricin and 600 μg/mL hygromycin. After incubation, the number of pink colonies were enumerated and efficiency calculated as [(number colonies/μg DNA) * (total volume μL/plated volume μL)] * dilution factor. Efficiencies for each dilution were averaged and these values for 3 independent experiments were reported as the mean ± SD.

### Biofilm assay.

Overnight cultures in YPD were washed three times by centrifugation with sterile PBS, counted by hemocytometer, and adjusted to 1 × 10^6^ cells/mL in 165 mM MOPS buffered RPMI 1640 medium, pH 7.0. Aliquots (100 μL) were transferred to cell culture-treated 96-well polystyrene plates and incubated at 37°C with gentle shaking for 24 h. The following day, wells were washed with sterile water and biomass stained by the crystal violet method as described previously (40).

### THP-1 cell challenge and immune activation.

THP-1 cells (THP1-null, Invivogen) were cultured according to the manufacturer’s protocol in RPMI 1640 medium plus l-glutamine supplemented with 10% heat-inactivated fetal bovine serum (HI-FBS), 25 mM HEPES, 100 U/mL penicillin-streptomycin, and 100 μg/mL normocin at 37°C and 5% CO_2_ as described previously (26). Cells were counted, seeded at 1 × 10^5^ cells per well of a tissue culture-treated 96-well polystyrene plate, and treated with 100 nM phorbol 12-myristate 13-acetate (PMA, Invivogen) for 24 h for differentiation into a macrophage phenotype. After aspirating culture medium, cells were challenged with 5 × 10^5^ C. albicans in fresh phenol-red free medium to generate a multiplicity of infection (MOI) of 5. Uninfected controls were also included. Cocultures were incubated for 4 h at 37°C, gently centrifuged to settle cells and fungi, and 100 μL of supernatant transferred to a 96-well plate containing an equal volume of prediluted 1× enzyme-linked immunosorbent assay (ELISA) assay buffer (Invitrogen) and assessed for IL-1β production using the human Ready-Set-Go! ELISA kit (Invitrogen). Results were reported as the mean + SD from 3 independent experiments.

### Hyphal growth assay.

Overnight cultures of C. albicans were washed three times in sterile PBS, counted on a hemocytometer, adjusted to 1 × 10^6^ cells/mL in buffered RPMI 1640, and incubated at 37°C with shaking at 200 rpm. At 4 h, aliquots were transferred to a glass slide, coverslipped, and cell morphology digitally captured by brightfield microscopy.

### Targeted amplicon sequencing and analysis.

Genomic DNA was isolated from 5 isolates each of Δ/Δ*ade2*, Δ/Δ*ade2*+*ADE2*, or mock transformed controls following overnight culture in YPD medium using the YeaStar genomic DNA kit according to manufacturer’s instructions (Zymo Research). Targeted PCRs were generated as follows: SC5314 (ADE2INTF and ADE2INTR), Δ/Δ*ade2* (ADE2INTF and ADE2INTR), Δ/Δ*ade2*+pDUP3-*ADE2* (NEUT5LAMPF and ADE2DETF, ADE2DETR and NEUT5LAMPR), and Δ/Δ*ade2*+pDIS3-*ADE2* (NEUT5LAMPF and ADE2DETR, ADE2DETF and NEUT5LAMPR). Amplicons were sent to Plasmidsaurus (plasmidsaurus.com) for sequencing on the Oxford Nanopore platform. Polished reads were returned as processed FASTA files. If necessary, sequences were aligned using SnapGene (4.1.9) software and manually merged at the point of PCR fragment overlap to generate a contiguous sequence. Experimental and expected sequences were aligned using blast+ (2.13), BAM files generated using samtools (1.15), and alignments visualized with JBrowse2 Desktop (1.6.9). A representative alignment for each strain was depicted and manually curated.

### Whole-genome sequencing and analysis.

Genomic DNA was isolated from 5 isolates each of Δ/Δ*ade2*, Δ/Δ*ade2*+*ADE2* (pDUP3), or mock transformed controls as described above. Library preparation and sequencing reactions were performed at the University of Alabama at Birmingham Heflin Center for Genomic Science. The Qiagen QIAseq FX DNA kit was used to prepare sequencing libraries using the manufacturer’s protocol and paired end 300 bp sequencing reads generated using the Illumina MiSeq platform. Bioinformatics analyses were provided by code4DNA (code4dna.com). Sequence reads from each sample were aligned using bwa mem (0.7.17) to the C. albicans reference genome build C_albicans_SC5314_version_A21-s02-m09-r10 downloaded from the Candida Genome Database (candidagenome.org) (41). Samtools (1.13) was used to sort alignment files based on genome coordinate and Picard (2.26.0) was used to mark duplicates in the sorted alignment files (42). Sequence variants were called using FreeBayes (1.1.0) with the diploid population-based model (43). GNU parallel was used to accelerate the freebayes step by dividing the eight chromosomes into multiple regions and then concatenated the results files using VCFtools (0.1.15) vcf-concat (44). The concatenated population-based model output vcf file was split into individual samples using VCFtools vcf-subset. Low quality (QUAL < 30) and low depth (DP < 10) variants were filtered out of each sample using VCFtools. Sequence variants for each sample were annotated using snpEff (4.5covid19). VCFtools vcf-isec was used to select mutations found in each affected sample but not in any sample from the corresponding control group. Alignments for each of the filtered sequence variants were visually inspected using JBrowse (1.15.2) and false positive variants were removed (45).

### Figure and graphic construction.

Images were constructed in GraphPad Prism (9.3.1), Microsoft Powerpoint (16.39), or Biorender (biorender.com). Any adjustments to brightness or contrast were applied evenly across images. All figures were processed as high-resolution images using Adobe Photoshop (21.1.1).

### Statistical analyses.

All data were plotted and analyzed for statistical significance using GraphPad Prism software. Data sets were tested for normality using a Shapiro-Wilks test. Data of multiple groups were compared using a one-way analysis of variance (ANOVA) and either Dunnett’s or Tukey’s posttests. Single or multiple Student’s t-tests were used for comparisons between two groups. Graphs were annotated to indicate significance levels (*, *P* < 0.05; **, *P* < 0.01; ***, *P* < 0.001).

### Data availability.

Whole-genome sequencing data files for C. albicans CRISPR-Cas9 altered and control strains have been deposited in NCBI SRA under the accession number PRJNA825548. Plasmid sequences were deposited in NCBI GenBank under the following accession numbers: pBSS2 (ON246334), CaHygB-flipper (ON287367), pDUP3-tADH1 (ON246333), pDIS3-tADH1 (ON246332).
