# Supplementary material for: Rapid Hypothesis Testing in Candida albicans Clinical Isolates Using a Cloning-Free, Modular, and Recyclable System for CRISPR-Cas9 Mediated Mutant and Revertant Construction
Source: Microbiol Spectr. 2022 May 25;10(3):e02630-21. doi: 10.1128/spectrum.02630-21 (PMC9241802; doi:10.1128/spectrum.02630-21)
Supplement: SUPPLEMENTAL FILE 1 — Supplemental material. Download spectrum.02630-21-s001.pdf, PDF file, 1.1 MB [file spectrum.02630-21-s001.pdf]

Oligonucleotide Name	Sequence (5' → 3')
**Vector construction**	
FLPPrACT1-F	TCAGTCGACATGCCACAATTTGATAT
PrACT1-R-PstI	TCA**CTGCAG**TTTGAATGATTATATTTTTTTAATATTAATATCGAGATAATGTTTC
CaPrMAL2-SEQF	GGCGATAGACGAAGACACGGTAT
FLP-SEQF	GTGACAGAGACAAAGACAAGCGTTA
SynthGene-F	CGCAGTTACGGATCAGTCAC
SynthGene-Rv2	CGCTACGATAGCTTACGATGATTC
CaPrACT1-DETF	GATCCATACACACACGTTAATTAGTTGTTCA
CaHygB-DETR	CAGCAGCAATAGCATCCATAGCT
UNIVCRISPR-R	GTGTGGAATTGTGAGCGGAT
CaHygB-SEQR	CCAAATGTCTAACTTCTGGACAATCTTCA
ADH1term-F-ClaI	TCA**ATCGAT**CGCGTGCATGCTAAGCAAATAG
ADH1term-R-SpeI	TGACA**ACTAGT**GAAAACTTGAAACTTGAAAACACCG
**crRNAs**	
crADE2up	GTACCGAGTCAAAATGTTTG
crADE2down	GGATAGATCTGTTAGAGATG
crALS3up	AGACCTCAATTCAAGGGAGG
crALS3down	ATCTAAAAAGGCGACTATGA
crCPH1up	GGATACTCTGTTGGTCAGGT
crCPH1down	TTATCTATGCATTATGTTTA

crECE1up	TAAAGCAAAAACAGTAGCAC
crECE1down	GGAATAAAAGATTAAGCTTG
crEFG1up	GGAATTTAATAAAACACGCC
crEFG1down	AATTTATGGCAGAAAGCAGA
crNEUT5pDUPup	GTAGTAAGACAATATGACTT
crNEUT5pDUPdown	TCAAGCACAATTCAGTTTTC
crNEUT5pDISup	GCTCGGAGGAGGCTCCCCAA
crNEUT5pDISdown	TGTTTAAATAATCAATTGAA

GENECC9KO Primers

ADE2CC9KO-F	TTTAGGCTTAGAGTGAAAAATCAATCCATCGCAGCAATAAAAAGAGGTTTTCCCAGTCACGACGT
ADE2CC9KO-R	GAATCAATGCTATTGAGGGGTTAGTTTTAAATGATAATTATAGTATTCACGTGTGGAATTGTGAGCGGAT
ALS3CC9KO-F	CATTTGCTAGACTTTCATGAATGTATATAAAAGAGGCTTCGGTTTTCCCAGTCACGACGT
ALS3CC9KO-R	TTTTGGAGCCAAAAAAACAAAAAACAAACAAATAACAAAAGTGTGGAATTGTGAGCGGAT
CPH1CC9KO-F	CATTTTCAACAAAGTATTGGATATAACACGTTTCTATCAATCATTAGGTTTTCCCAGTCACGACGT
CPH1CC9KO-R	AACCAAGTATATATTCCTTTAAAAATAAATCATAATAAAACAAGTGTGTGGAATTGTGAGCGGAT
ECE1CC9KO-F	AACAAACAACTTTCCTTTATTTTACTACCAACTATTTTCCATTCGTTAAAGGTTTTCCCAGTCACGACGT
ECE1CC9KO-R	AAAACAACAATTAAAAAAATCAGTTACAGCAAAAGTGTCACAAGACTTATGTGTGGAATTGTGAGCGGAT
EFG1CC9KO-F	TTCCAAGAGTTAATTGATTAAACAACTTGGTCCAAGAATTCATTAGGTTTTCCCAGTCACGACGT
EFG1CC9KO-R	TTTCCAATCATTTGTTAATGAAATATATGCTATAATCTAATTTGGGTGTGGAATTGTGAGCGGAT

GENEINT Primers

FLPINTF (universal)	CGCGCGTAATACGACTCACT
FLPINTR (universal)	CAAGCGCGCAATTAACCCTC
ADE2INTF	CTAAGCTAGTCGTGATGGTTAATATTTTTGCT
ADE2INTR	GGTTGGAGAGATTCTCGATCTCAAGTAT
ALS3INTF	CGTGCATAAGAAAGTTTTGCTATGC
ALS3INTR	CTGAACAATTCATAGTCGCAGGC
CPH1INTF	CTTCTGTCCACACACACACTACCA
CPH1INTR	ACGCAGCTTAGATGGGGTTG
ECE1INTF	GCCCGCCCACAAATCTTAC
ECE1INTR	CCAGACGTTGGTTGCAAAACTTAAAC
EFG1INTF	GTGTATTACATCCAGCCAACCCAC
EFG1INTR	CTTGGTACTTGATGTCTTATGCATCACC

GENEDET Primers

ADE2DETF	GCTCATGTTCGTGCTGTCAC
ADE2DETR	CTATGAGGAGTTCTGTGTGCAC
ALS3DETF	ACTGTCACCGCTCCACC
ALS3DETR	TCACCTGGAGGAGCAGTGATT
CPH1DETF	GGCAGATGCCTTGGAACGAGA
CPH1DETR	GGGTAGTGGAGTCCTTATTGGTTGG
ECE1DETF	GAAGATATTGATTCTGTTGTTGCTGG

ECE1DETR	CAGTTTCCAGGACGCCAT
EFG1DETF	CAACTGCAGCCAATCAACAGG
EFG1DETR	CAGCAGTAGTGGCAGCCTTG
SAT1DETF	GTGCAAGGCAATTGATTCAACATTGC
CaHygBDETF	GGTTGGCTTGTATGGAACAACAAACT

Revertant construction primers

NEUT5homology-pDUPF (universal)	GCAGATATGAGATAAAAGTTTTAAAGGACAAGAAAAGG
NEUT5homology-pDISF (universal)	GGAGGCTCCCCAAAGATTTTATCA
Nt5ADH1upUNIVOL-R (universal)	CGATACCGTCGACCTCGAGG
ADH1tUNIVOL-F (universal)	CGCGTGCATGCTAAGCAAAT
NEUT5homology-pDUPR (universal)	ATCTCTAATAATTGCAATTGCAATTGCTTCACATA
NEUT5homology-pDISR (universal)	CGATTCGTGTTTAAATAATCAATTGAAAAGAAAATAG
ADE2PrOL-F	*CCTCGAGGTCGACGGTATCG*GCAAATCATTATAGGGCGTGGG
ADE2OL-R	*ATTTGCTTAGCATGCACGCG*TGCACCATAACGTTTACTTGTTTAATATGCT
ALS3PrOL-F	*CCTCGAGGTCGACGGTATCG*CTCGCAGACTAACTGAACCAAGAA
ALS3OL-R	*ATTTGCTTAGCATGCACGCG*ACCCTGAAACTGTACAAGCGAT
ECE1PrOL-F	*CCTCGAGGTCGACGGTATCG*GGTTTAGCACGGTCTGAACTCTTT
ECE1OL-R	*ATTTGCTTAGCATGCACGCG*GCTTGTGGAAAACAAATTTTTATCTGCTGA
NEUT5LAMPF (universal)	GCTGAATCACTTGATAGGATTTAGTTCCATTATGG
NAT1INTF (universal)	CCCAGATGCGAAGTTAAGTGCG
NEUT5LAMPR (universal)	GGAATTTCTAGTCACTTGACACGACC

PrADE2INTR	GTGCCACTGATCAGCAGCTGA
PrALS3INTR	CCGTTTTTGCAATGGGTTCTATCATCA
PrECE1INTR	TGCTCGTGTAATTTGATGATGAGGAATG
NAT1DETR	GCATCACCTGGAACAGAAGTTC
ADE2SEQR2	CTAATGATCTGACAACCATCACAGCC
ALS3SEQF1	GTTCCTGCCGGTTATCGTCCA
ALS3SEQF3	CAACCACAACTGTAACTGCACCA
ALS3SEQF4	CGCCTATCATTTCTTCTAGTGCTGAT
ALS3SEQR	GCAAGTGGTAAAGTGACAGTACCCA
ADH13SEQR (universal)	ATATCGCACTCACGTAAACAC

Table S1. Oligonucleotides used in this study for plasmid and strain construction and validation. Bolded only sequences indicate restriction enzyme sites. Underlined only sequences indicate regions that bind to *SAT1*- and *CaHygB*-flipper plasmids. Sequences italicized and underlined indicate reverse complementarity to Nt5ADH1upUNIVOL-R and ADH1tUNIVOL-F primers.

sample	chromosome	position	gene	variant type	reference	change
S6	4	1413726	C4_06370C	missense	T	C
S7	1	2333435	GCA2	missense	G	A
	R	290585	CDC27	stop gained	G	C
S8	1	2266527	RPA34	cons. in frame insertion	TTCCTCCTCCTCCT	TTCCTCCTCCTCCTCCT
	2	2060461	C2_10050W	missense	G	C
S9	1	2333435	GCA2	missense	G	A
	4	1413726	C4_06370C	missense	T	C
S10	R	290585	CDC27	stop gained	G	C

Table S2. Variants identified in the SCΔ/Δ*ade2*+*ADE2* but not SCΔ/Δ*ade2* groups. Sequence reads were aligned to the SC5314 reference genome, sequence variants called using FreeBayes, and variants called using VCFtools. Variants with quality scores < 30 and depth < 10 were filtered out of the analysis. Average read depth per sample was 50X coverage.

**Strain**	**Parent**	**Genotype**	**Reference**
SC5314	--	wild-type	(1)
SCΔ/Δ*ade2*HygNAT	SC5314	Δ*ade2*:*SAT1* Δ*ade2*:*CaHygB NEUT5L*/*NEUT5L*	this study
SCΔ/Δ*ade2*	SC5314	Δ*ade2*:FRT+ Δ*ade2*:FRT+ *NEUT5L*/*NEUT5L*	this study
SCΔ/Δ*ade2*+*ADE2*	SCΔ/Δ*ade2*	Δ*ade2*:FRT+ Δ*ade2*:FRT+ Δ*neut5l*:Pr*ADE2*-*ADE2*-t*ACT1*/*NEUT5L*	this study
SCΔ/Δ*ece1*	SC5314	Δ*ece1*:FRT+ Δ*ece1*:FRT+ *NEUT5L*/*NEUT5L*	this study
SCΔ/Δ*ece1*+*ECE1* (pDUP)	SCΔ/Δ*ece1*	Δ*ece1*:FRT+ Δ*ece1*:FRT+ *NEUT5L*/Pr*ECE1*-*ECE1*-t*ADH1*/*NEUT5L*	this study
SCΔ/Δ*ece1*+*ECE1* (pDIS)	SCΔ/Δ*ece1*	Δ*ece1*:FRT+ Δ*ece1*:FRT+ Δ*neut5l*:Pr*ECE1*-*ECE1*-t*ADH1*/*NEUT5L*	this study
Δ/Δals3	SC5314	Δ*als3*:FRT+ Δ*als3*:FRT+ *NEUT5L*/*NEUT5L*	this study
Δ/Δals3+ALS3	Δ/Δals3	Δ*als3*:FRT+ Δ*als3*:FRT+ Δ*neut5l*:Pr*ALS3*-*ALS3*-t*ACT1*/*NEUT5L*	this study
Δ/Δ*cph1*	SC5314	Δ*cph1*:FRT+ Δ*cph1*:FRT+	this study
Δ/Δ*efg1*	SC5314	Δ*efg1*:FRT+ Δ*efg1*:FRT+	this study
Δ/Δ*cph1* Δ/Δ*efg1*	Δ/Δ*cph1*	Δ*cph1*:FRT+ Δ*cph1*:FRT+ Δ*efg1*:FRT+ Δ*efg1*:FRT+	this study
529L	--	wild-type	(2)
529LΔ/Δ*ade2*	529L	Δ*ade2*:FRT+ Δ*ade2*:FRT+ *NEUT5L*/*NEUT5L*	this study
529LΔ/Δ*ade2*+*ADE2*	529LΔ/Δ*ade2*	Δ*ade2*:FRT+ Δ*ade2*:FRT+ Δ*neut5l*:Pr*ADE2*-*ADE2*-t*ACT1*/*NEUT5L*	this study
529LΔ/Δ*ece1*	529L	Δ*ece1*:FRT+ Δ*ece1*:FRT+ *NEUT5L*/*NEUT5L*	this study
529LΔ/Δ*ece1*+*ECE1*	529LΔ/Δ*ece1*	Δ*ece1*:FRT+ Δ*ece1*:FRT+ Δ*neut5l*:Pr*ECE1*-*ECE1*-t*ACT1*/*NEUT5L*	this study
JS15	--	wild-type	this study
JSΔ/Δ*ade2*	JS15	Δ*ade2*:FRT+ Δ*ade2*:FRT+ *NEUT5L*/*NEUT5L*	this study
JSΔ/Δ*ade2*+*ADE2*	JSΔ/Δ*ade2*	Δ*ade2*:FRT+ Δ*ade2*:FRT+ Δ*neut5l*:Pr*ADE2*-*ADE2*-t*ACT1*/*NEUT5L*	this study
JSΔ/Δ*ece1*	JS15	Δ*ece1*:FRT+ Δ*ece1*:FRT+ *NEUT5L*/*NEUT5L*	this study
JSΔ/Δ*ece1*+*ECE1*	JSΔ/Δ*ece1*	Δ*ece1*:FRT+ Δ*ece1*:FRT+ Δ*neut5l*:Pr*ECE1*-*ECE1*-t*ACT1*/*NEUT5L*	this study
SJCA1	--	wild-type	this study
SJCAΔ/Δ*ade2*	SJCA1	Δ*ade2*:FRT+ Δ*ade2*:FRT+ *NEUT5L*/*NEUT5L*	this study
SJCAΔ/Δ*ade2*+*ADE2*	SJCAΔ/Δ*ade2*	Δ*ade2*:FRT+ Δ*ade2*:FRT+ Δ*neut5l*:Pr*ADE2*-*ADE2*-t*ACT1*/*NEUT5L*	this study
SJCAΔ/Δ*ece1*	SJCA1	Δ*ece1*:FRT+ Δ*ece1*:FRT+ *NEUT5L*/*NEUT5L*	this study
SJCAΔ/Δ*ece1*+*ECE1*	SJCAΔ/Δ*ece1*	Δ*ece1*:FRT+ Δ*ece1*:FRT+ Δ*neut5l*:Pr*ECE1*-*ECE1*-t*ACT1*/*NEUT5L*	this study
TW1	--	wild-type	(3)
TWΔ/Δ*ade2*	TW1	Δ*ade2*:FRT+ Δ*ade2*:FRT+ *NEUT5L*/*NEUT5L*	this study
TWΔ/Δ*ade2*+*ADE2*	TWΔ/Δ*ade2*	Δ*ade2*:FRT+ Δ*ade2*:FRT+ Δ*neut5l*:Pr*ADE2*-*ADE2*-t*ACT1*/*NEUT5L*	this study
TWΔ/Δ*ece1*	TW1	Δ*ece1*:FRT+ Δ*ece1*:FRT+ *NEUT5L*/*NEUT5L*	this study
TWΔ/Δ*ece1*+*ECE1*	TWΔ/Δ*ece1*	Δ*ece1*:FRT+ Δ*ece1*:FRT+ Δ*neut5l*:Pr*ECE1*-*ECE1*-t*ACT1*/*NEUT5L*	this study

Table S3. Strains constructed or used in this study.

1. Gillum AM, Tsay EY, Kirsch DR. 1984. Isolation of the *Candida albicans* gene for orotidine-5'-phosphate decarboxylase by complementation of *S. cerevisiae* ura3 and *E. coli* pyrF mutations. Mol Gen Genet 198:179-182.
2. Rahman D, Mistry M, Thavaraj S, Challacombe SJ, Naglik JR. 2007. Murine model of concurrent oral and vaginal *Candida albicans* colonization to study epithelial host-pathogen interactions. Microbes Infect 9:615-622.
3. White TC. 1997. Increased mRNA levels of *ERG16, CDR, and MDR1* correlate with increases in azole resistance in *Candida albicans* isolates from a patient infected with human immunodeficiency virus. Antimicrob Agents Chemother 41:1482-1487.

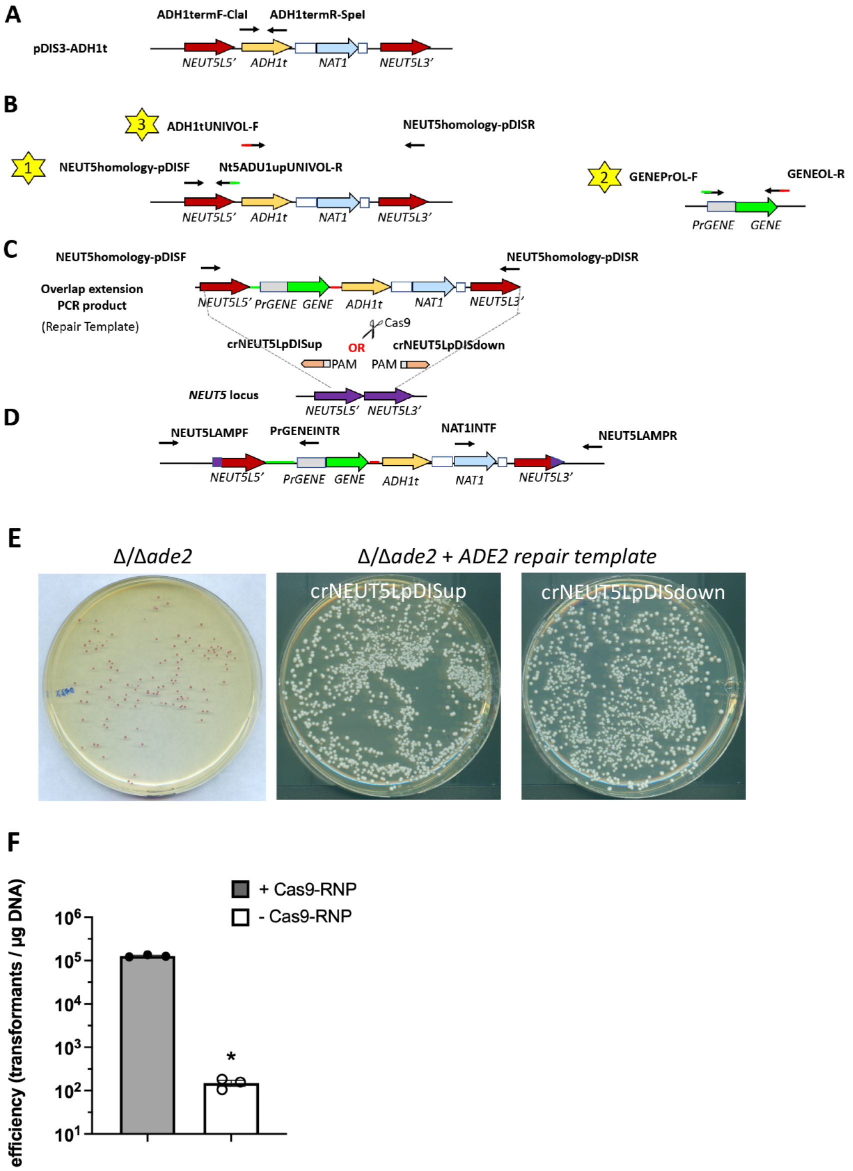

Figure S1. A cloning-free and CRISPR-Cas9-mediated approach to gene reversion at the *C. albicans NEUT5* locus by homologous recombination. (A) Plasmid pDIS3 was modified to contain the *ADH1* terminator sequence to generate plasmid pDIS3-tADH1. **(B)** Overlap extension PCR is utilized to fuse 3 PCR fragments generated by independent reactions (yellow stars) using either pDIS3-tADH1 (reactions 1 and 3) or *C. albicans* genomic DNA (reaction 2) as templates. Primers contain conserved overlap sequences depicted as red and green lines. **(C)** The fused cassette is integrated at the *NEUT5* neutral locus by CRISPR-Cas9 transformation in *C. albicans*

using either crNEUT5LpDISup or crNEUT5LpDISdown crRNAs and contains the *NAT1* marker conferring resistance to nourseothricin. **(D)** The integrated cassette is depicted. Due to position of 5' and 3' *NEUT5* homology arms, it integrates in the forward orientation. Primers NEUT5LAMPF and PrGENEINTR and NAT1INTF and NEUT5LAMPR are used to detect correct integration at the *NEUT5* locus by PCR. **(E)** The Δ/Δ*ade2* mutant was transformed using the above approach with a wild-type copy of *ADE2*. Digital imaging of YPD plates containing 200 µg/mL NAT depicts reverted white colonies. Images are representative of 3 independent repeats. **(F)** Transformation efficiency of pDIS3-ADE2 was calculated with (gray box) and without (white box) Cas9-RNP. Data is depicted as the mean ± SD of 3 independent experiments. Statistical significance was assessed using a Student's t-test. *, $p < 0.05$.

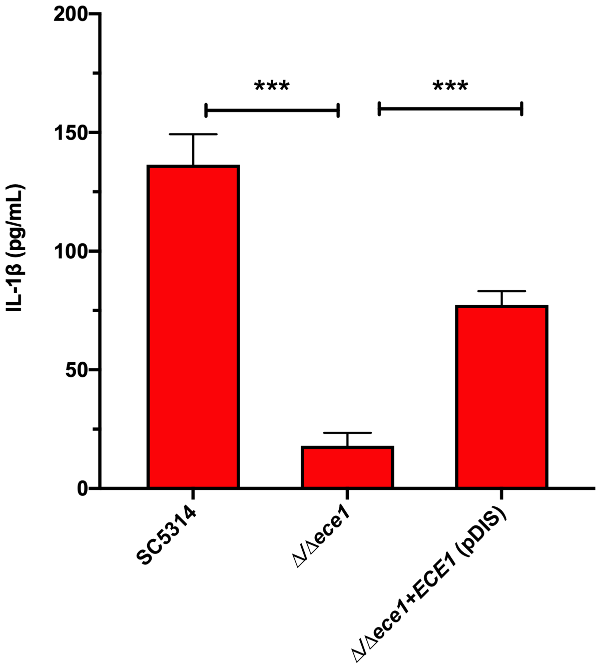

Figure S2. Reversion of *ECE1* with a pDIS3-tADH1 repair template similarly restores pathogenicity. THP-1 cells were challenged with SC5314 *C. albicans* wild-type, mutant, and revertant (created with pDIS3-tADH1) strains at an MOI of 5 for 4 h. Culture supernatants were assessed for the inflammatory cytokine IL-1β by ELISA. Data is depicted as the mean + SD from 3 independent experiments. Statistical significance was assessed by one-way ANOVA and Dunnett's post-tests. *** $p < 0.001$.
